# Hyperthermophile Protein Behavior: Partially-Structured Conformations of *Pyrococcus furiosus* Rubredoxin Monomers Generated through Forced Cold-Denaturation and Refolding

**DOI:** 10.1371/journal.pone.0080014

**Published:** 2014-03-06

**Authors:** Sanjeev Kumar Chandrayan, Satya Prakash, Shubbir Ahmed, Purnananda Guptasarma

**Affiliations:** 1 Department of Biological Sciences, Indian Institute of Science Education & Research (IISER) Mohali, Knowledge City, Sector-81, SAS Nagar (Mohali), Punjab, India; 2 Protein Science & Engineering Division, Institute of Microbial Technology, (IMTECH), Council of Scientific & Industrial Research (CSIR), Chandigarh, India; Aligarh Muslim University, India

## Abstract

Some years ago, we showed that thermo-chemically denatured, partially-unfolded forms of *Pyrococcus furiosus* triosephosphateisomerase (PfuTIM) display cold-denaturation upon cooling, and heat-renaturation upon reheating, in proportion with the extent of initial partial unfolding achieved. This was the first time that cold-denaturation was demonstrated for a hyperthermophile protein, following unlocking of surface salt bridges. Here, we describe the behavior of another hyperthermophile protein, the small, monomeric, 53 residues-long rubredoxin from *Pyrococcus furiosus* (PfRd), which is one of the most thermostable proteins known to man. Like PfuTIM, PfRd too displays cold-denaturation after initial thermo-chemical perturbation, however, with two differences: (i) PfRd requires considerably higher temperatures as well as higher concentrations of guanidium hydrochloride (Gdm.HCl) than PfuTIM; (ii) PfRd's cold-denaturation behavior during cooling after thermo-chemical perturbation is incompletely reversible, unlike PfuTIM's, which was clearly reversible (from each different conformation generated). Differential cold-denaturation treatments allow PfRd to access multiple partially-unfolded states, each of which is clearly highly kinetically-stable. We refer to these as ‘*Trishanku*’ unfolding intermediates (or TUIs). Fascinatingly, refolding of TUIs through removal of Gdm.HCl generates multiple partially-refolded, monomeric, kinetically-trapped, non-native ‘*Trishanku*’ refolding intermediates (or TRIs), which differ from each other and from native PfRd and TUIs, in structural content and susceptibility to proteolysis. We find that the occurrence of cold denaturation and observations of TUI and TRI states is contingent on the oxidation status of iron, with redox agents managing to modulate the molecule's behavior upon gaining access to PfRd's iron atom. Mass spectrometric examination provides no evidence of the formation of disulfide bonds, but other experiments suggest that the oxidation status of iron (and its extent of burial) together determine whether or not PfRd shows cold denaturation, and also whether redox agents are able to modulate its behavior.

## Introduction

We reported some years ago [1] that the hyperthermophile protein, *Pyrococcus furiosus* triosephosphate isomerase (PfuTIM), displays inordinately high kinetic stability and some very intriguing conformational behavior upon exposure to heat and the denaturant, guanidium hydrochloride (Gdm.HCl). When the protein is heated to temperatures approaching, or exceeding, 100°C in the presence of Gdm.HCl, it undergoes partial unfolding to a degree dependent on the concentration of Gdm.HCl, and the temperature up to which heating is done (e.g., 95, 100 or 105°C); this dependence is clearly seen in the changes evident in the protein's far-UV CD spectrum.Rather unexpectedly, cooling of the heated Gdm.HCl-containing protein solution back to 25°C did not result inany refolding of the partially-unfolded PfuTIM; instead, further partial unfolding was observed. We established beyond all reasonable doubt that this further unfolding owed to cold denaturation. PfuTIM samples prior to heating were thus demonstrably different in structural content from the very same samples after they were subjected to heating and cooling (with absolutely no attendant aggregation), even though the two samples were otherwise identical in all respects, in regard to their physical and chemical environments. There was no evidence of these differently-structured samples showing any inter-conversion over the time scale of experimentation. In summary, PfuTIM's differential conformational responses to heating and cooling in the presence of denaturant, considered together with the non-coincidence of the protein's initial and final conformational states at 25°C, after heating and cooling, thus clearly indicated that no facile equilibrium exists between different conformers in this hyperthermophile protein, unlike what is generally seen in proteins derived from mesophiles. The tendency of the protein to be trapped kinetically in a variety of different conformational states thus suitably explained the previously notedhigh kinetic thermal stability of PfuTIM's native structure [2].

PfuTIM has a 225 residues-long polypeptide chain, and is associated into a tetrameric quaternary structure [2]. To explore whether the unusual conformational behavior seen with PfuTIM can also be observed in a much smaller protein of hyperthermophile evolutionary origin, we carried out the same treatment on a very small protein, the well-known and widely studied 53 residues-long, iron-sulphur cluster-containing protein, rubredoxin (PfRd) from *P. furiosus* [3], [4]. PfRd is one of the most kinetically thermostable proteins known to man [5],[6], with an unfolding rate of 1.9×10^−6^ s^−1^, at pH 7.0 and 100°C. Several excellent studies exploring the thermal stability of PfRd and, in particular, its kinetic thermal stability have been described in the literature [5],[6],[7],[8],[9]. Another thing that caused us to study this protein is the fact that it contains a disproportionately large number of charged residues. Of the 53 amino acid residues in PfRd, 18 are charged residues (34%) representing only 3 of the 20 naturally-occurring amino acids (5 lysines, 7 aspartates, and 6 glutamates) which should have normally had a representation of only 15% assuming proportional representation of each amino acid. Clearly, charged residues are playing an important role in PfRd. Since we reported that in PfuTIM the observed effects owe primarily to surface salt-bridges, it became imperative that we examine a small hyperthermophile protein like PfRd, with disproportionately high numbers of charged residues. Furthermore, what made PfRd interesting was the fact that it contains a small iron-sulfur cluster and also a tight aromatic cluster in its interior which we believe (based on unpublished observations which have already been communicated for publication), to be crucially linked to iron atom binding and formation of the correct native structure.

We report here that the behavior of PfRd, despite its small size, is even more intriguing and complex than the behavior of PfuTIM. Whereas PfuTIM's cooling-induced partial unfolding had been found to be reversible to the exact extent to which it had occurred [1], [2], for a given degree of heating (in a given concentration of Gdm.HCl), PfRd's cooling-induced partial unfolding was found to be incompletely reversible, unlike what was seen with PfuTIM. This allowed successive cycles of re-heating and re-cooling in denaturant to unfold the molecule to progressively greater extents, facilitating isolation and study of what appear to be different monomeric, kinetically-trapped structural intermediate populations, generated either through partial thermo-chemical unfolding and subsequent cold denaturation, or through refolding of the partially-unfolded states following removal of Gdm.HCl. Fascinatingly, we learned that PfRd's ability to display such behavior owes to the redox status of its bound iron atom. The presence and oxidation status of the iron determines whether or not this small protein is able to behave like a hyperthermophile protein capable of displaying different unfolding/refolding intermediates. Within cold-denatured states, the iron is buried within a tightly packed structure which is inaccessible to redox agents such as beta-mercaptoethanol. However, at high temperature in the presence of denaturant, the cold-denatured forms lose structure sufficiently to provide the redox agent access to the buried oxidized iron, leading to its reduction. Protein containing reduced iron behaves just like any other mesophile protein.

## Materials and Methods

### Recombinant PfRd

A synthetic gene encoding PfRd was created through splicing by overlap extension PCR (SOE-PCR) using overlapping synthetic oligos. DNA sequences are described below for six overlapping oligonucleotides, numbered F1, R1, F2, R2, F3 and R3, that alternate between the gene's two strands to cover its entire span. Three separate splicing reactions were performed using three independent sets of primers, F1-R1, F2-R2 and F3-R3, as megaprimers. The products of the F1-R1 and F2-R2 reactions were then spliced, and amplified, through use of F1 and R2 as primers. This reaction's product was further spliced to the product of F3-R3, and the whole construct amplified using F1 and R3 as primers. These reactions incorporated the restriction sites Nde I and Xho I at the 5′ and 3′ ends of the gene. The sequences of the oligonucleotides described above were as follows :

**F1**-(5-TATACTATGGCGCAACATATGGCTAAATGGGTTTGCAAAATCTGT-3′);

**R1**-(5′-GTCACCAGCGTCTTCGTCGTAGATGTAACCACAGATTTTGCAAACCC-3′);

**F2**-(5′-CGAAGACGCTGGTGACCCTGACAATGGCATCTCCCCGGGTACCAAA-3′);

**R2**-(5′-GACAAACCCAGTCGTCAGGCAGTTCTTCGAATTTGGTACCCGGGGA-3′);

**F3**-(5′-TGACGACTGGGTTTGTCCGATCTGTGGTGCTCCAAAATCCGAATTT-3′);

**R3**-(5′-CGCACTATCCTCGAGGTCTTCCAGTTTTTCAAATTCGGATTTTGGAG-3′).

The synthesized PfRd-encoding gene (sequencing data shown in Figure S1 in File S1) was digested with the enzymes, Nde I and Xho I, and cloned into pET23-a for expression as a recombinant protein possessing a C-terminal 6×His affinity tag in the expression host *E.coli* BL21DE3pLysS. Transformed *E.coli* cells were grown in 1 litre 2×TY cultures, induced at O.D (600 nm) of ∼0.5 with 1 mM IPTG at 37°C, and lysed under non-denaturing conditions for Ni-NTA affinity chromatography-based purification by standard protocols recommended by the Ni-NTA resin manufacturer (Qiagen). The purified protein was red in color, indicating that it had folded and incorporated the iron-sulphur cluster. It may be noted that no exposure of the protein to heat was necessary at any stage. The amino acid sequence of the produced PfRd along with its pI, predicted extinction coefficient, and other characteristics, may be viewed in Figure S2 in File S1, together with an SDS-PAGE showing the homogeneity of the purified PfRd. It may be noted that the above recombinant clone possessed a C-terminal 6×His affinity tag. We have also similarly created a clone with an N-terminal 6×His tag and observed no difference in behavior whatsoever.

### Chemicals

Guanidium hydrochloride (GdmCl), 2-mercaptoethanol and all other chemicals were obtained from Sigma Chemical Co. or GE Healthcare (USB Chemicals), USA.

### Thermo-chemical denaturation and spectroscopic monitoring

PfRd in 20 mM phosphate buffer (pH 7.2), at a concentration of ∼0.10–0.25 mg/ml was used for all experiments, with or without 4M or 6 M Gdm.HCl. A quartz cuvette of dimensions 1 mm (path length) ×10 mm×40 mm was used for monitoring changes in circular dichroism (CD) signal on a Jasco J-810 spectropolarimeter with an inbuilt Peltier (RTE) attachment, and a 9 mm metal spacer block to facilitate rapid heat transfer for real-time measurements involving changes in temperature. The block's temperature was measured with an internal probe located close to the cuvette. Heating and cooling of protein solutions was carried out using rates of change of temperature of 3°C, 2°C, or 1°C per minute, with ellipticity data at 222 nm being simultaneously measured with a bandpass of 4 nm, a response time setting of 16 seconds, and a standard sensitivity scale of 100 millidegrees (mdeg). Far-UV CD spectra between 250–210 or 250–190 nm were collected through averaging of five spectral scans, using nitrogen flowing at 30 litres/min, from a Peak Scientific NM30 gas generator. For fluorescence studies involving protein emission from aromatic groups, excitation was done at 280 nm and emission scanned in the region of 300–400 nm, at 25°C. For monitoring the emission of 8-anilino-1-naphthalenesulfonate (ANS) bound to the protein, excitation was done at 355 nm, with emission scanning done between 380–550 nm. For measuring the absorption spectra of PfRd, a Varian Cary-50 spectrometer was used. We also examined the effects of including a reducing reagent in the heating and cooling reactions, because of the possibility that disulfides could form amongst the four proximally situated cysteine residues upon removal of the coordinated iron atom during heat unfolding in the presence of Gdm.HCl. Briefly, we included 2-mercaptoethanol at a concentration of 14.3 mM at various stages of heating and cooling, e.g., before the initial heating, after the initial heating but before the initial cooling, or after the initial cooling. We measured the CD signal to examine the effect, if any, of reduction of any disulfide bonds formed.

### Chromatographic and electrophoretic studies

Gel filtration chromatography was performed on a Bio-Rad Biologic Duoflow instrument, using a Bio-Sil SEC 250 column, for comparison of the hydrodynamic volume of PfRd with that of the refolding intermediates. The separation range of this column is 10,000–3,00,000 Da, and the nominal bed volume of the column is 12 ml. This makes it ideal for distinguishing between monomeric PfRd and higher-order oligomers, since the monomer has a molecular weight of ∼7.0 kDa which is just below the resolving range of the column. Monomers would thus elute close to the bed volume of ∼12.0 ml Oligomers, including dimers, would be expected to elute in the fractionation range which is optimally stretched out at elution volumes close to the bed volume of the column. Native-PAGE electrophoresis was performed using a pH of 8.8 in the stacking and resolving gels, and 17% acrylamide, primarily to examine whether PfRd molecules remain monomeric and also to detect how much of the original protein sample remains after proteolytic treatment

### Proteolysis and mass spectrometry

Native PfRd and refolded PfRd intermediates (20 µl of 0.25 mg/ml) were subjected to digestion by subtilisin, or trypsin, using molar ratios of PfRd∶protease of 100∶1. Reactions were stopped by mixing the sample with MALDI matrix CHCA (10 mg/ml solutions of pH∼2.0). The products of proteolysis were visualized by native-PAGE, and detected by MALDI-TOF mass spectrometry (see Figures S3 and S4 in File S1), which allowed monitoring of masses generated through proteolysis as well as time points at which different masses were generated.

### Examination of the effect of redox agents on PfRd's conformational behavior

A beta-mercaptoethanol (B-ME) concentration of 14.3 mM was used to examine the effect of redox agents on PfRd's conformational behavior during heating and cooling in the presence of Gdm.HCl (i.e., during thermo-chemical perturbation of structure) to examine the effect of B-ME on the cold-denaturation and heat-renaturation behavior of PfRd. The rationale for doing this was to explore whether either of two possibilities was affecting the observations, namely : (i) the formation of disulfides between cysteine residues upon departure of iron from the binding site, or (ii) the formation of a tightly-folded non-native structure around the bound iron atom, effectively limiting the ability of the whole molecule to undergo facile unfolding or refolding. In the first case, the expectation was that B-ME would reduce the disulfide, if present. In the second case, the expectation was that B-ME would reduce the iron, if accessible. Heating and cooling cycles used temperature variation at a rate of 3°C/min and 6M Gdm.HCl. The maximum temperature used was 98°C or 100°C, using the same protein concentration of PfRd (0.138 mg/ml). The effect of B-ME on PfRd's conformation was monitored through observation of the far-UV CD signal at 222 nm, in a Jasco J-810 spectropolarimeter,with temperature being maintained by its in-built peltier, as previously described in another section of the materials and methods.

### Cysteine modification and mass spectrometry

Several protocols have been published for detection of free or bound cysteines using cysteine modification [10], [11], [12]. In most, iodoacetamide is used as cysteine modifying agent in the presence and absence of a reducing agent. Modified cysteines are then detected using mass spectrometric analysis,though the observation of an increase in mass of 57 Da owing to each site of modification, with respect to the control peak(s) derived for intact protein masses (where the protein being modified is small) or of tryptic peptides derived from the protein (where the protein is large). In all experiments reported in this paper, iodoacetamide (IA, Sigma-A3221) was used as cysteine modifying (alkylating) agent and beta-mercaptoethanol (BME) was used as the reducing agent. IA powder was dissolved in 600 µL milliQ water to give 500 mM stock concentration. The working concentrations of IA and 2-mercaptoethanol used were 40 mM and 10 mM respectively. The buffer used was 20 mM sodium phosphate buffer, pH 7.2. PfRd has four cysteines bound to iron. In PfRd, a hyperthermophile protein, cysteines are only accessible at high temperature in presence of 6M Gdm.HCl (control experiments for different temperatures are shown in the Supplementary data; the relevant figure citations are provided later). To modify the cysteines, PfRd was incubated with IA and B-ME (presence or absence, as required) at 90°C or above for 10 minutes in the presence of 6M Gdm.HCl (20 mM sodium phosphate buffer, pH 7.2). The resulting solution was desalted using reverse phase Zip-TipC18 tips (Millipore) and eluted samples were analysed using MALDI-TOF. For detection of disulfides in various cold-denatured and heated/cooled states of PfRd, the following was done. All samples had final concentrations of 0.5 mg/ml of PfRd and 6M Gdm.HCl. Two types of samples were made. S1 samples containing PfRd and 6M Gdm.HCl with no heat treatment, and S2 samples containing PfRd and 6M Gdm.HCl, with heating done up to 100°C and cooling done back to 25°C. Heating and cooling of samples was done in an Eppendorf, Mastercycler (pro S) using a ramp rate of ∼3.6°C/minute. S1 samples were used to detect disulfide formation during the first heating and cooling cycle, while S2 samples were used to detect disulfides already formed during a previous heating and cooling cycle. For detection of disulfides during and after cold denaturation a series of experiments were performed which had different steps of thermal treatment (i.e., different temperatures) and chemical treatment (e.g., addition of B-ME or IA). After elution from Zip-TipC18 tips, samples were analysed by MALDI-TOF mass spectrometry and examined for increase in expected mass of ∼57 Da.

## Results and Discussion

Given the small size of PfRd, it would indeed be quite remarkable for it to show cold-denaturation (and related) behavior similar to that of PfuTIM, which is considerably bigger in size and also different in quaternary structural status. There is also the question of differences existing between the behavior of iron-bound PfRd (holo-PfRd) and iron-lacking PfRd (apo-PfRd). Interestingly, apo-PfRd (produced by unfolding PfRd completely, and chelating away the released iron atom) is known to no longer display hyperthermal stability [13]. It behaves like any ordinary mesophile protein, displaying facile melting in the absence of any denaturant at a temperature of ∼70°C, as well as melting in the presence of 6 M Gdm.HCl, in the absence of any heating. Separately, in another paper (not yet in print; unpublished observations), we have demonstrated that there are differences in the details of packing of six aromatic residues forming a cluster in the interior of PfRd, between iron-bound PfRd (holo-PfRd) and apo-PfRd produced by refolding the PfRd chain in the absence of iron (Apo-1 PfRd). We have shown that these differences could be responsible for the profound differences in the apparent (kinetic) stabilities of the two forms. We have also shown that if the iron atom is teased out from holo-PfRd, without performing any unfolding or refolding, to produce a differently-obtained apo form called Apo-2 PfRd, this latter apo form displays the extreme hyperthermal stability of the holo form, rather than the meso-stability of the Apo-1 form. It is not possible to reintroduce iron into either apo form. Thus, we would like to preface the discussion of the results presented below by emphasizing (in work that is not yet published) that we have established that the hyperthermal stability of PfRd owes to a set of intramolecular interactions (primarily involving an aromatic cluster in the protein's interior) that occur in a manner dependent on the binding of an iron atom at some early time point during folding of PfRd *in vivo*.

Certainly, therefore, it is necessary to take into account the cold-denaturation behavior of holo-PfRd. One possibility that especially needs to be taken into account is what could happen to PfRd upon removal of its iron atom. The iron atom is coordinated by four cysteine residues that are mutually distally located in the sequence, but proximal to each other in the molecule's three-dimensional structure. If removal of the iron atom during thermochemical unfolding were to facilitate any formation of disulfide bonds amongst these cysteine residues, certainly this could complicate the interpretation. Therefore, we decided to explore PfRd's behavior separately in the presence, and absence, of a reducing reagent such as beta-mercaptoethanol (BME), to look for differences in behavior. However, with a view to exploring the entire gamut of the molecule's behavior under non-reducing conditions [i.e., conditions allowing either disulfide bond formation and/or the retention of the oxidized state by a bound, and buried, iron atom in an oxidized state] before performing comparisons with what occurs under reducing conditions, we decided to proceed and finish studying PfRd's conformational behavior during thermo-chemical unfolding and cooling, from all conceivable angles.

Therefore, all our studies (and data obtained) can be divided into two sections, the first presenting studies conducted under non-reducing conditions and the second presenting studies conducted under reducing conditions in the presence of beta-mercaptoethanol. In each sub-section below, we clarify at the outset whether the data was obtained under non-reducing or reducing conditions, proceeding to integrate and synthesize the two types of data into a unified view towards the end of the paper.

### Non-reducing conditions: Different extents of thermo-chemical unfolding of PfRd are attained with differentramping rates, denaturant concentrationsand temperatures

The effects of temperature and denaturant on the structure of PfRd were examined by heating and cooling the protein [∼0.10–0.25 mg/ml; in 20 mM phosphate, pH 7.2] in the presence of various concentrations [4 M or 6 M] of Gdm.HCl, at different temperature ramping rates [1°C, 2°C, or 3°C] and by performing heating and cooling between different sets of temperature points[25°C–95°C, 25°C–100°C or 25°C–103°C].

**Figure 1:**
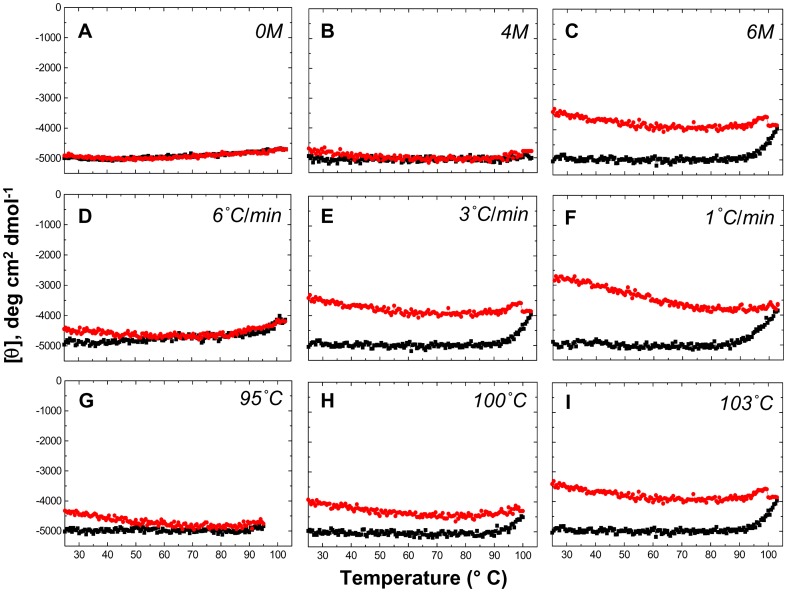
TUI data. Changes in PfRd's mean residue ellipticity CD signal at 222 nm monitored as a function of increasing (black curve) and decreasing (red curve) temperatures, in combination with one of the following three additional variables : (i) varying concentrations of Gdm.HCl (*Panels A–C*), (ii) varying rates of heating (*Panels D–F*), and (iii) varying degrees of heating, i.e, up to different temperatures (*Panels G–I*). The values of the variables used to collect the data for each panel are mentioned as insets in the relevant panels.

Figure 1 shows that there is only a negligible (reversible) change in MRE when PfRd is exposed to temperatures ranging from 25°C to 103°C alone (Figure 1A), or to temperatures in this range in the presence of 4 M Gdm.HCl (Figure 1B). A profound effect is seen, however, when the concentration of Gdm.HCl is increased to 6 M (Figure 1C). At this Gdm.HCl concentration, there is a significant reduction in negative MRE upon raising of temperature to 92–93°C and above. Notably, reversal of the rise in temperature through cooling back to lower temperatures does not reverse the change that was initially seen in the negative MRE signal, from −5000 to −4000 deg cm^2^ dmol^−1^; instead, there is a further reduction in MRE, from −4000 to −3200. In this respect, the behavior of PfRd mimics that of PfuTIM, in that initial thermo-chemical perturbation allows the molecule to display cold-denaturation, which is not seen otherwise as a consequence of temperature changes alone in the absence of denaturant, or in the presence of insufficient denaturant. Our original paper on PfuTIM's cold denaturation [1], may be referred to for a full and detailed analyses of why hyperthermophile proteins do not ordinarily display cold denaturation, but can be persuaded to do so when surface electrostatic interactions are destabilized by thermo-chemical denaturation involving a denaturant that is also an electrolyte.

Keeping the concentration of Gdm.HCl fixed at 6 M, we changed the rate of ramping of temperature from 3°C/min (Figure 1E; reproduced from Figure 1C, for comparison) to 6°C/min (Figure 1D). The reduction observed in the negative MRE signal was now clearly much lower than that obtained with 3°C/min, as well as somewhat reversible. This may be explained by the fact that the protein is given less of a cumulative duration of exposure to high temperatures when a faster ramping rate is used, such that there is less scope for the protein to undergo similar extents of partial unfolding. In line with this interpretation, we found that when the rate of temperature ramping is instead reduced from 3°C/min to 1°C/min (Figure 1F), this gives the sample a much greater cumulative duration of exposure to high temperatures, which correspondingly results in significantly greater degrees of initial thermo-chemical unfolding (from −5000 to −3600) as well as a much greater degree of further unfolding observed upon cooling (down to −2750), occurring presumablythrough cold-denaturation. The difference in behavior at different ramping rates is clearly indicative of some kinetic thermal structural stability.

We next decided to hold the ramping rate fixed at 3°C/min, and the Gdm.HCl concentration also fixed at 6 M, to change only the temperature up to which heating was done. We found that heating to a relatively low temperature like 95°C elicits almost no observable initial thermo-chemical perturbation (Figure 1G). However, despite this, some loss of MRE signal still occurs (down to −4400) upon cooling of the sample, indicating that subtle changes have occurred to allow some cold-denaturation. Of course, when heating is done to a higher temperature like 100°C (Figure 1H), or to an even higher temperature like 103°C (Figure 1I; identical to Figures 1C and 1E, reproduced for comparison), the extent of the structural effects is increased.

**Figure 2:**
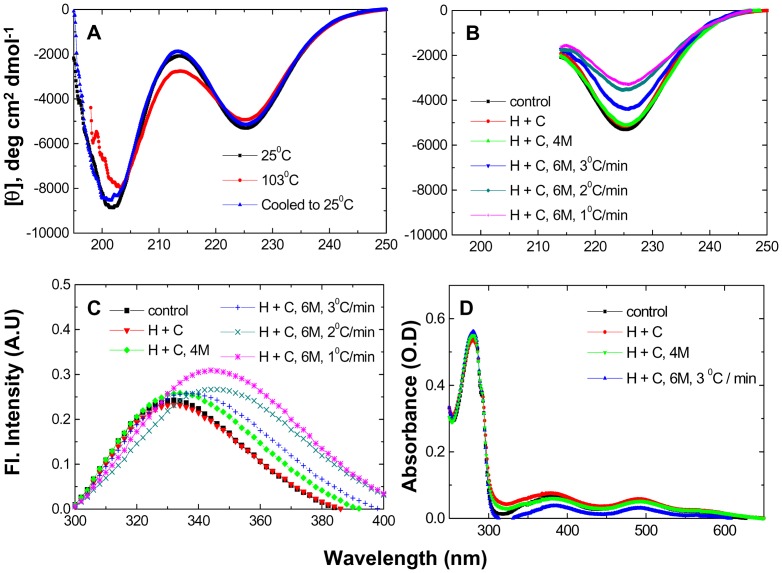
TUI data. Spectral changes in PfRd as a function of heating (H) and cooling (C) in the presence of denaturant. Individual values of temperatures, Gdm.HCl concentrations, rates of heating, and manners of treatment applicable to each panel/curve are mentioned as insets within panels. H+C indicates that the sample has been heated from 25°C to 103°C, and cooled back to 25°C. *Panel A* : Control far-UV CD spectra of PfRd in the absence of Gdm.HCl, before, after heating, and after cooling. *Panel B* : Far-UV CD spectra of PfRd collected at 25°C, before heating, and after heating and cooling using different treatments, as mentioned. *Panel C* : Fluorescence emission spectra of PfRd collected at 25°C, before heating, and after heating and cooling using different treatments, as mentioned, corresponding to samples for which CD spectra are shown in Panel B. *Panel D* : UV-visible absorption spectra of PfRd collected at 25°C, before heating, and after heating and cooling, using different treatments as mentioned, corresponding to some of the samples shown in Panels B and C, and a representative spectrum for a sample using a lower Gdm.HCl concentration.

In terms of requisite controls, Figure 2A shows the far-UV CD spectra of PfRd at 25°C in the absence of Gdm.HCl, at 103°C after heating of the sample at 3°C/min, and at 25°C following cooling of the sample back to 25°C at the same rate. A further set of control and experimental CD spectra, all collected at 25°C, is shown in Figure 2B. Data is shown for (a) PfRd at 25°C prior to heating, (b) PfRd after heating to 103°C and cooling back to 25°C in the absence of denaturant (reproduced from Figure 2A, for comparison), and (c) PfRd after heating to 103°C and cooling back to 25°C in the presence of 4 M and 6 M Gdm.HCl. This set of spectra establishes that spectral changes signifying alterations in structure are only seen in the PfRd samples heated to 103°C and cooled back to 25°C in the presence of 6 M Gdm.HCl using different rates of temperature ramping (3°C/min, 2°C/min, and 1°C/min). None of the other samples show any changes. The trend in the reduction of band intensities shows that greater extents of partial unfolding are obtained by using slower rates of temperature ramping. In summary, therefore, Figures 1 and 2 show that there are no effects (on PfRd's conformation) when heating and cooling are done in the absence of denaturant (i.e., PfRd undergoes no thermal unfolding), but that there are clear and differential effects of combining heat and denaturant in different ways, demonstrating that PfRd's native conformation is vulnerable to undergoing different extents of partial thermo-chemical unfolding, both during heating in the presence of denaturant and during subsequent cooling in the presence of denaturant.

In support of the CD data in Figure 2B, fluorescence emission spectral data are presented in Figure 2C for essentially the same samples. Here too, it may be noted that there are changes in the wavelengths of maximal fluorescence emission (i.e., ^em^λ_max_ values), from ∼330 nm to 336 nm, and further to 345 nm, signifying alterations in the local environments of the molecule's two Trp residues; again these changes are seen only in PfRd samples heated to 103°C and cooled to 25°C in the presence of 6 M Gdm.HCl, and not when heating/cooling is performed in the absence of denaturant. As with the CD spectra, different ramping rates produce different extents of change in the ^em^λ_max_, demonstrating that reduction of temperature ramping rateselicits greater exposure of Trp residues and, thereby, greater red-shifting of ^em^λ_max_ values to longer wavelengths.

The data in the CD and fluorescence spectra shown in Figures 2A–C are further supported by changes in the molecule's absorption spectra (Figure 2D). A reduction in the intensity of the absorption bands at 390 nm, and 490 nm, signifies changes in the iron-sulphur cluster owing to structural perturbations, either because of physical loss of iron or due to the reduction of iron upon conformational alteration, owing to its microenvironment. Such reduction in absorption is only seen in samples heated and cooled in 6 M Gdm.HCl, i.e., only in samples subjected to thermo-chemical unfolding.

To summarize all of the above data: (A) PfRd is found to be an extraordinarily highly stable protein, requiring 6 M Gdm.HCl and heating at temperatures approaching, or exceeding, 100°C in order to undergo even partial loss of structure. (B) PfRd loses structure further upon cooling from high temperature, indicating that chain segments that have undergone initial thermo-chemical perturbation may have become vulnerable to limited unfolding (of perturbed regions) through a cold-denaturation process akin to that seen previously in PfuTIM, as reported by us earlier. (C) Clearly, the high stability of PfRd owes at least partly to kinetic factors, because the rate of temperature ramping influences the extent of structural change seen. (D) The most important thing to note is that PfRd, a small 53 residues-long protein, can exist in multiple different conformational states subject to the same extant physical and chemical conditions, depending on the history of the protein's treatment prior to its being placed in those conditions.This is rather unprecedented, for such a small protein, although similar behavior has been observed by us with PfuTIM. We would like to refer to these states as *Trishanku* unfolding intermediate states (TUIs), as each state appears to be a kinetically-trapped state which is neither natively-folded nor completely unfolded.

However, in respect, of the last observation above, i.e., item D, at least in principle, it remains possible that some of the observations could be caused, or influenced, by adventitious disulfide bond formation accompanying removal of the iron atom, or reduction of the bound iron atom. This aspect is explored later. Meanwhile, as shown in Figures 2B, 2C and 2D, we emphasize that the structural characteristics of PfRd in 6 M Gdm.HCl at 25°C can range widely, from completely folded protein to significantly unfolded protein forms, with no inter-conversion amongst these forms being apparent or visible over the timescales of these experiments, or even over much longer timescales (of weeks, or months; as examined for a few samples). This suggests that kinetics, and not thermodynamics, is the most important determinant of PfRd's structure over the experimental and observational timescales used. This also suggests that only some parts, or regions, of the protein which have been released from such kinetically-stable stateshave become free to come under purely thermodynamic control of conformation, such that they are able to exhibit cold-denaturation upon lowering of temperatures below 102–103°C (otherwise not seen).

That a protein of such a small size should display such suggestive evidence of regional autonomy in its structure (for maintenance of its folded state) would be fascinating, even if adventitiously formed disulfide bonds were to play a role, notwithstanding the contention that it is difficult to explain how disulfide bonding alone could facilitate the adoption of such a multiplicity of states. In any case, we present evidence later in the paper that there are no disulfide bonds formed. This makes the observations even more fascinating.

### Non-reducing conditions: Repetitive cycles of heating and cooling of PfRd in Gdm.HCl lead to successively greater degrees of unfolding, due to irreversible cold-denaturation

Once a certain amount of initial thermo-chemical perturbation has been achieved by heating samples to a particular temperature in the presence of Gdm.HCl, it would be interesting to heat and cool the sample repetitively to examine what happens to its conformation. With PfuTIM, it had previously been seen that the profiles of changes re-traced themselves, over and over again. Thus, re-heating of a partially cold-denatured sample of PfuTIM in denaturant was found to essentially reverse the cold-denaturation achieved, to the exact extent to which it had been achieved originally, in each experiment. Also, cooling of such a sample after heating was observed to once again bring about the same extent of cold-denaturation that was originally generated in that sample [1].

**Figure 3:**
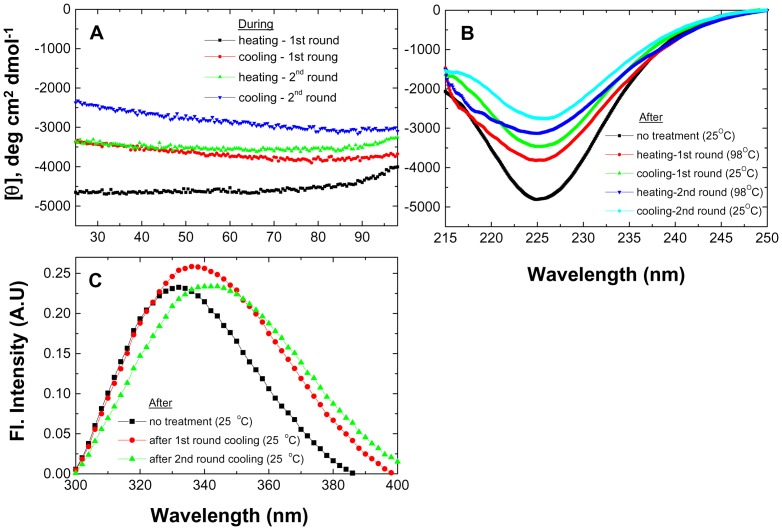
TUI data. Spectral changes in PfRd in the presence of 6°C/min. Parameters describing individual treatments and temperatures applicable to each curve are mentioned alongside as inset within the relevant panels. *Panel A* : Changes in PfRd's mean residue ellipticity CD signal at 222 nm as a function of heating and cooling, monitored during two successive rounds of heating and cooling of the same sample. *Panel B* : Far-UV CD spectra of PfRd collected at different temperatures, before heating, after heating, and after cooling, through two successive rounds of heating and cooling, corresponding to Panel A. *Panel C* : Fluorescence emission spectra of PfRd collected at 25°C, before heating, and after the first, and second, rounds of heating and cooling.

In contrast, PfRd behaved very differently as described below; again, it is noted that this part of the study did not explore whether disulfide bonding had occurred through removal of the iron atom; the effects of including reducing agents are described in later sections which are clearly marked with the words, ‘reducing conditions’, in the sub-title. What is observed with PfRd is that re-heating of cold-denatured samplesbetween 25°C and 98°Ccauses further partial thermo-chemical unfolding, instead of reversing the cold-denaturation. This is shown for PfRd samples heated in 6 M Gdm.HCl in Figure 3A. Likewise, re-cooling of such re-heated samples achieves an even greater degree of cold-denaturation. Our interpretation of this difference in the behavior of PfuTIM and PfRd is that PfuTIM is a much larger protein and, therefore, more likely than PfRd to show a high degree of kinetic stability in its various sub-structures. Thus, following the initial thermo-chemical perturbation achieved, any segments of PfuTIM which happen to become unfolded through cold-denaturation do not elicit any further unfolding, and merely return to the state from which they were derived, without affecting any other parts or segments of the protein. In other words, small sections (e.g., supersecondary structural motifs such as beta-alpha units) within PfuTIM could be conformationally autonomous of other sections to such an extent that there is little interdependence between parts and, therefore, a lower chance that any part unfolded through cold-denaturation would influence any neighboring part to also cooperatively undergo unfolding.

In contrast, in PfRd which is much smaller, it is likely that a higher degree of cooperativity (or lower degree of insulation of any substructure from neighboring substructures) could be seen, with less scope for conformational autonomy of behavior of substructures. Therefore, any segment undergoing some conformational change in PfRd would be more likely to affect neighboring segments. Thus, even though the lower degree of regional autonomy in PfRd does not extend to the protein's displaying any cooperative global unfolding (like any ordinary mesophile protein), evidently each bit of partial unfolding of PfRd predisposes other neighboring bits to unfold to a greater extent.

To further establish what is shown in (and suggested by) Figure 3A, Figure 3B shows changes in the 225 nm negative band within the far-UV CD spectra of PfRd samples, using temperature and denaturant conditions identical to those used in various stages of the experiment shown in Figure 3A, i.e., at 25°C, at 98°C, at 25°C after cooling from 98°C, at 98°C after re-heating of the cooled 25°C sample, and at 25°C after re-cooling. The non-overlap of all of these spectra in Figure 3B shows that the various samples all differ in their structural contents. Such behavior is unprecedented in proteins. Without invoking segmental unfolding and kinetically-stable partially-unfolded structures, it is impossible to explain either how, or why, either the three spectra collected at 25°C do not overlap with each other, or indeed why the two spectra at 98°C do not overlap. Certainly, studies of mesophile proteins have led us to believe that a protein population's conformational characteristics must be identical for measurements made under identical physical and chemical conditions, assuming purely thermodynamic control of conformation. That even a small hyperthermophile protein like PfRd fails to follow this dictum over these experimental timescales is highly intriguing.

Figure 3C shows the fluorescence emission spectra of PfRd samples at 25°C from three different stages of the experiment shown in Figures 3A and 3B: i.e., before the initial heating, after the first round of heating and cooling, and after the second round of heating and cooling. It is clearly seen that there is a progressive red-shifting of the ^em^λ_max_ from 330 nm to 336 nm, and on to 342 nm, once again demonstrating the existence of structural non-equivalence amongst samples subject to the same physical and chemical conditions during measurement. The control set of fluorescence spectra, in this regard, have already been shown in Figure 2C.

### Non-reducing conditions: Different conformations of PfRdresult from removing of Gdm.HClfrom samples subjected to different conditions of thermo-chemical unfolding

The effects of heating to 98°C, or 103°C, and cooling of samples back to 25°C, in the presence of 6 M Gdm.HCl, have already been described for experiments using different temperature ramping rates of 3, 2 and 1°C/min. As was shown, use of a ramping rate of 1°C/min causes PfRd to adopt a substantially-unfolded state. We performed extensive dialysis of such a sample heated to 103°C and cooled using a ramping rate of 1°C/min, to examine its structural state after complete removal of Gdm.HCl (designated sample I, or TRI-I).

Separately, to examine the effect of excessive exposure to high temperatures in the presence of denaturant, we performed an additional experiment in which PfRd was heated to 103°C and incubated at this temperature for 1 hour, prior to being cooled back to 25°C. This cooled sample (designated sample II, or TRI-II) was also dialyzed extensively, to completely remove Gdm.HCl.

**Figure 4:**
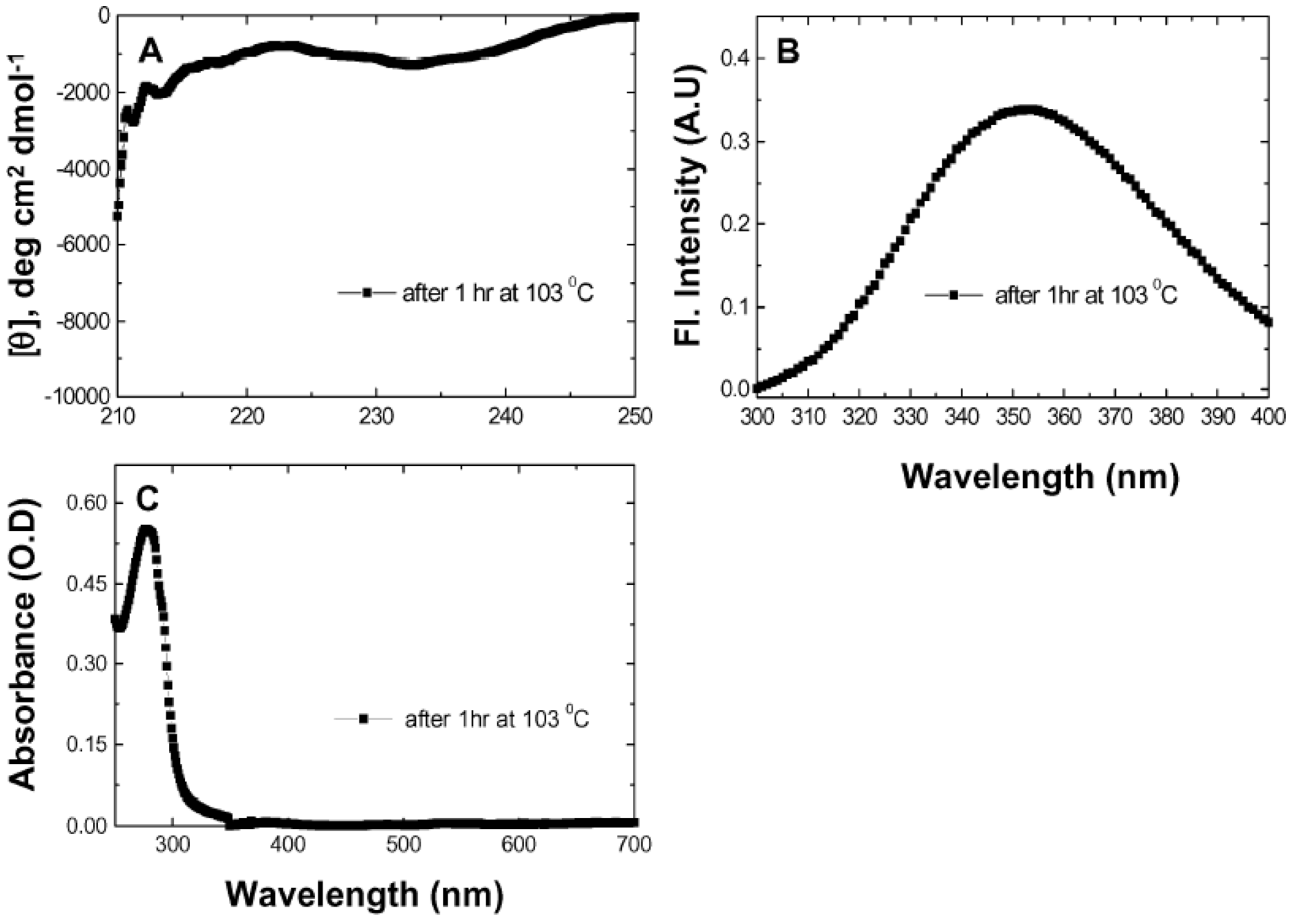
TUI data. Spectral changes in PfRd monitored after incubation at 103°C for 1 hour in the presence of 6 M Gdm.HCl, and cooling to 25°C. *Panel A* : Far-UV CD spectrum. *Panel B* : Fluorescence emission spectrum. *Panel C* : UV-visible absorption spectrum.

We describe below first the differences seen between samples I and II, before dialysis, before moving on to the description and analyses of the two samples, after dialysis. The CD, fluorescence, and absorption data for undialyzed sample I have already been presented in Figures 2B, 2C and 2D respectively. For undialyzed sample II which has not been described hitherto, Figure 4A presents the CD spectrum, while Figures 4B and 4C, respectively, present the fluorescence and absorption data. The CD spectrum shows that the negative 225 nm band is completely destroyed, giving rise to a minor negative band at ∼232 nm, in undialyzed sample II (Figure 4A). The fluorescence data shows that undialyzed sample II has an ^em^λ_max_ of 352 nm (Figure 4B), indicating that its Trp residues are completely exposed to the aqueous solvent, in contrast to native PfRd which has an ^em^λ_max_ of ∼330 nm, indicating buried Trp residues. The UV-visible absorption data reveals that the 390 and 490 nm bands deriving from PfRd's iron-sulphur cluster are no longer present in undialyzed sample II (Figure 4C). It is evident from Figures 4A–C, therefore, that PfRd's structure is nearly completely destroyed through 1 hr of incubation at 103°C although, of course, the persistence of some residual structure cannot be altogether ruled out.

It may be concluded, therefore, that undialyzed sample II represents a nearly completely-unfolded Trishanku unfolding intermediate of PfRd, whereas undialyzed sample I represents a partially-unfolded Trishanku unfolding intermediate of PfRd. We took these two profoundly different samples of PfRd, and removed Gdm.HCl. This was done to examine whether any refolding occurs, and also to examine the extent to which they differ from each other, and from native PfRd, after removal of Gdm.HCl.

**Figure 5:**
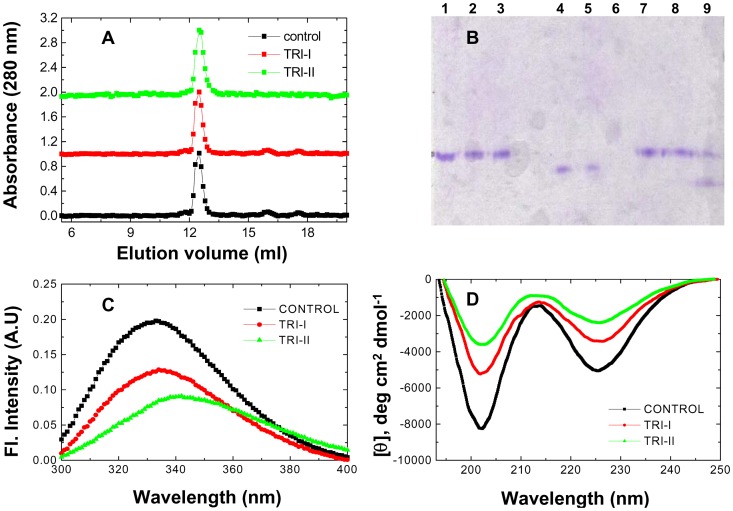
TRI data. Comparison of the characteristics of native PfRd (control) with the characteristics of two non-native PfRd states, TRI-I and TRI-II, obtained through dialysis-based removal of Gdm.HCl from PfRd samples, following (for the TRI-I sample) heating 1°C/min to 103°C at and cooling to 25°C at the same rate, and separately (for the TRI-II sample) incubation at 103°C for 1 hour, followed by cooling to 25°C. *Panel A* : Gel filtration chromatograms on Bio-Sil SEC-250 columns. *Panel B* : Native (17%) PAGE of native PfRd (lane 1), TRI-I (lane 2), TRI-II (lane 3); subtilisin-treated samples of native PfRd (lane 4), TRI-I (lane 5), TRI-II (lane 6); and trypsin-treated samples of native PfRd (lane 7), TRI-I (lane 8), TRI-II (lane 9). *Panel C* : Fluorescence emission spectra of native (control) and TRI-I and TRI-II states of PfRd. *Panel D* : Far-UV CD spectra of native (control) and TRI-I and TRI-II states of PfRd.

The first thing that we examined was, of course, whether there was any aggregation. No aggregation could be seen. Gel filtration chromatography (Figure 5A) revealed that both dialyzed samples I and II are monomeric like native PfRd. Neither sample showed evidence of the presence of any exposed hydrophobic patches, in relation to control PfRd, to the extent that this could be assessed through ANS binding studies (data not shown). It may also be noted here that the resolution of the column is insufficient to detect any differences in conformation and hydrodynamic volume between different conformers, allowing distinctions to be made only between monomers and higher association states. We next ran the samples on native-PAGE (Figure 5B). Fascinatingly, the dialyzed forms of sample I (lane 2), and sample II (lane 3),appear to have exactly the same mass-charge characteristics as the control native PfRd sample (lane 1), since the three samples show identical mobility. As the native PfRd is known to be monomeric, the Native-PAGE data once again confirms the monomeric nature of the dialyzed forms of samples I and II. Below, we describe the results of structural-biochemical examination of these samples.

Fluorescence spectroscopy shows that dialyzed sample I has an ^em^λ_max_ of 336 nm (Figure 5C), in comparison to undialyzed sample I (Figure 2C) which had an ^em^λ_max_ of ∼343 nm. This shows that some refolding occurs in sample I upon removal of Gdm.HCl through dialysis, since there is clearly substantial burial of the molecule's Trp residues evidenced by the blue-shift of 7 nm in the ^em^λ_max_ from 343 to 336 nm.This conformational change is not reflected, however, in either the shape of the CD spectrum, or the intensity of the 225 nm negative MRE band in undialyzed sample I (Figure 2B), which remains close to −3250 deg cm^2^ dmol^−1^, even after removal of Gdm.HCl (Figure 5D).

Likewise, fluorescence spectroscopic examination shows that there is also some refolding in sample II upon removal of Gdm.HCl. The undialyzed form of sample II containing 6 M Gdm.HCl had an ^em^λ_max_ of ∼352 nm (Figure 4B), whereas the dialyzed form has an ^em^λ_max_ of 342 (Figure 5C), showing that the molecule's Trp residues which were previously completely exposed to the aqueous solvent become somewhat buried through partial refolding accompanying removal of Gdm.HCl.

In the case of sample II, there is also a very profound change in the shape of the CD spectrum, as well as in the intensity of the 225 nm negative MRE band (Figure 5D), compared to undialyzed sample II (Figure 4B). The MRE value at 225 nm, which is only about −850 deg cm^2^ dmol^−1^ in undialyzed sample II increases to about −2100 upon dialysis, along with the regeneration of the characteristic 225 nm negative band. It appears from Figure 5D that this dialyzed sample II has refolded to acquire roughly half of the structural content of the native PfRd.

CD and fluorescence studies thus establish that dialyzed samples I and II differ profoundly from each other, and from their erstwhile states before dialysis, in structural-biochemical terms – even though clearly both are monomers, like native PfRd, in the absence of Gdm.HCl. We probed the differences in their structures by probing the structures for susceptibility to cleavage by one non-site-specific protease, subtilisin, and one site-specific protease, trypsin. The data is shown in Figure 5B. Lanes 4, 5 and 6 represent equal amounts of control (native) PfRd, dialyzed sample I, and dialyzed sample II, respectively, following exposure of all three to subtilisin using protease∶protein molar ratios of 1∶100. Whereas dialyzed sample I survives almost to the same extent as control PfRd, dialyzed sample II appears to be totally degraded. The time course of degradation was monitored by MALDI-TOF mass spectrometry (Figure S3 in File S1). Lanes 7, 8 and 9 of Figure 5B are the equivalent of lanes 4, 5 and 6, except that trypsin was used, instead of subtilisin, with the same protease∶protein molar ratios, and identical durations of incubation. Again, dialyzed sample I was found to resemble control PfRd, whereas dialyzed sample II was substantially digested, releasing a trypsin-resistant fragment.

To summarize the data presented in this section, dialysis clearly leads to substantial refolding, both in the initially partially-unfolded (Trishanku) unfolding intermediate subjected to refolding (sample I, or TRI-I) and in the nearly completely-unfolded (Trishanku) unfolding intermediate (sample II, or TRI-II) subjected to refolding, with both remaining monomeric after refolding, and with sample I resembling native PfRd in terms of its susceptibility to proteolysis. Here, the word ‘Trishanku’ simply denotes an entity stuck between two extreme and opposite states, i.e., between fully-unfolded and fully-folded states. From all structural-biochemical viewpoints, both forms are intermediates that are unable to transform into either native PfRd or unfolded PfRd. Thus, they may be thought of as Trishanku refolding intermediates (TRI) of PfRd, TRI-I and TRI-II. The dialyzed (refolded) samples I and II, i.e., TRI-I and TRI-II were heated to 102°C at 3°C/min, to examine the extent to which they retain the thermal stability of native PfRd. Especially with sample II (which had been nearly completely unfolded, prior to refolding through removal of Gdm.HCl), we wished to see whether the refolded form, TRI-II, displays any thermal stability at all.

**Figure 6:**
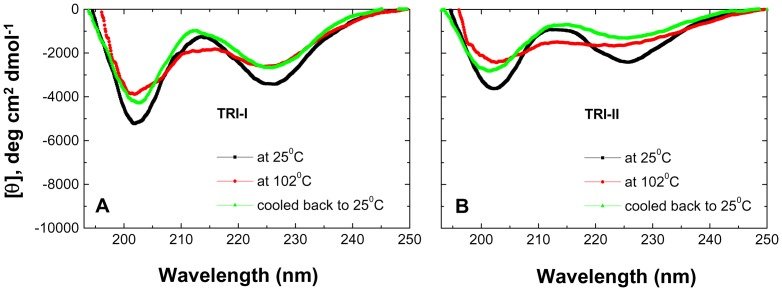
TRI data. Comparison of temperature-dependent changes in the far-UV CD spectra of TRI-I (*Panel A*) and TRI-II (*Panel B*). The values of the temperatures at which data was collected are mentioned within the panels as inset.

Figure 6A shows the entire far-UV CD spectra of TRI-I at 25°C, upon heating to 102°C, and after cooling back to 25°C. It is clearly seen that exposure to 102°C does not lead to complete unfolding of TRI-I, but only to some marginal structure loss. Interestingly, upon heating, the 225 nm and 203 nm band intensities are both reduced, while negative MRE intensity of the inter-band maximum at 212–213 nm is increased. When cooling is effected, the 225 nm and 203 nm band intensities remain almost the same as at 102°C, i.e., they do not return to the original 25°C values, but the intensity of the 212–213 nm inter-band maximum returns to the original 25°C value. The same effect is seen in an even more profound manner with TRI-II (Figure 6B); however, the net change is higher for TRI-II as would indeed be expected, because it was unfolded to a greater extent to begin with and also refolded back to a state farther away from native PfRd than TRI-I. Figure S5 in File S1 presents supporting data in the form of temperature (heating/cooling) CD traces of TRI-I and TRI-II, monitoring MRE at ∼222 nm. These results also support the same conclusion, namely that TRI-I and TRI-II are also hyperthermostable, like PfRd.

It may be mentioned here, in passing, that for several of these samples we incubated the samples for months, and in one case for a whole year, and failed to observe any further changes, suggesting that the various TUI and TRI states are truly, and effectively, kinetically-trapped. Also, it may be noted that some representative control experiments involving simple time-traces and spectra of control incubations of PfRd were done in the presence of 6 M Gdm.HCl at two different temperatures, The same are shown in Figure S6 in File S1 which shows time-traces and spectra for incubations at 100°C, while Figure S7 in File S1 shows time-traces and spectra for incubations at 20°C.

### Reducing conditions: Addition of B-ME inhibits cold denaturation of thermo-chemically perturbed PfRd

**Figure 7:**
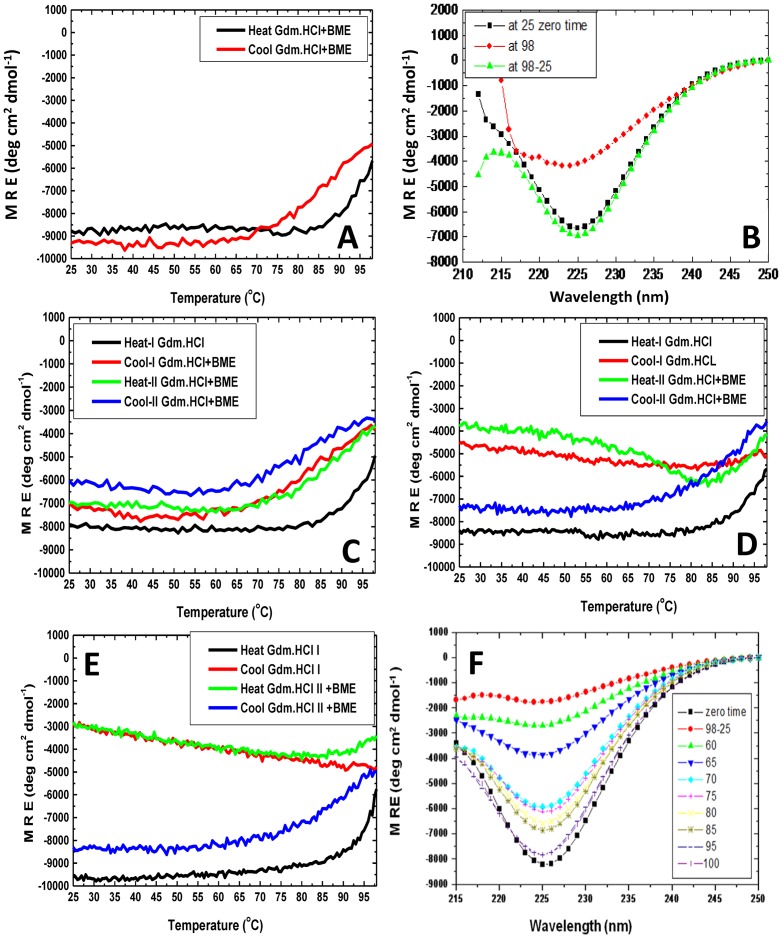
Cold denaturation behaviour of PfRd; its reversibility and the effect of B-ME. *Panel A*: Cold denaturation in PfRd was inhibited by B-ME added at 25°C before initial heating (black curve, 25°C). *Panel B* :Reversal of cold denaturation, observed through monitoring of reversal in far-UV CD spectral features (secondary structural content). Black curve shows the spectrum of the native form in the presence of Gdm.HCl and B-ME prior to heating; the red curve shows the spectrum at 98°C after first heating; the green curve shows the spectrum at 25°C after cooling from 98°C.*Panel C* : Cold denaturation in PfRd reversed by B-ME added at 98°C after first heating (black curve, 98°C). No further cold-denaturation (blue curve) seen; instead cold-denaturation is reversed. *Panel D* : Cold denaturation in PfRd reversed by B-ME added at 25°C. The first heating-cooling cycle is shown by the black (heating) and red (cooling) curves, showing cold-denaturation. Then B-ME was added. The second heating cycle is shown by the green (heating) and blue (cooling) curves, and B-ME is seen to have abrogated cold-denaturation and returned the protein to original state. *Panel E* :In contrast to the curves in panel D, where B-ME was added at 25°C, here B-ME was added at 98°C, after the second heating. Again, only after B-ME is added, is the reversal of cold-denatuation seen. *Panel F* : Cold denaturation in PfRd can be reversed significantly by B-ME when added after the first heating-cooling cycle, but only if the temperature at which it is added is above 65–70°C. The black curve is the spectrum of native PfRd and the red curve is the spectrum of cold-denatured PfRd. All other curves represent PfRd's spectrum after heating to a certain temperature (mentioned in the panel), adding B-ME, and cooling back to room temperature. It is seen that if B-ME is added at temperatures below 70°C, the spectrum does not return to that of native PfRd. Otherwise, the spectrum returns to that of PfRd.

We discovered that the commonly used reducing agent, beta-mercaptoethanol (B-ME), which is known to reduce disulphide bonds in proteins and also reduce oxidized iron in metallurgical applications, effectively and substantively inhibits the cold denaturation of PfRd. As described in the above sections, in the absence of B-ME, thermo-chemical perturbation of PfRd leads to the display of cold denaturation by sections of the protein's structure. Therefore, to examine the effect of B-ME, we carried out the initial thermo-chemical perturbation in the presence of B-ME (at a concentration of 14.3 mM; i.e., a 1000-fold dilution of pure B-ME which has a molarity of 14.3 M). Under these conditions, we found that instead of displaying cold denaturation, PfRd instead displayed a tendency to refold during cooling in the presence of B-ME. Figure 7A displays the profile plotting changes in mean residue ellipticity (MRE) with temperature. Figure 7B plots the 225 nm negative MRE band at different temperatures, and it is evident from this figure that PfRd heated and cooled in Gdm.HCl containing B-ME returns to the original folded state. It is already known that PfRd lacking bound oxidized iron behaves in a very different manner from native holo-PfRd; in such a state, the protein behaves very much more like any other mesophile protein of ordinary structural stability, having lost all of its extraordinarily stable character [13]. Thus, one possible explanation for the above data is that the B-ME reduces iron and physically removes it from PfRD. Alternatively, the iron could still be present bound in a reduced state, in its original pocket or elsewhere in the protein. In any case, B-ME appears to alter the protein's behavior to cause it to become akin to that of iron-lacking PfRd created by fully unfolding PfRd and refolding it in the absence of iron (apo-1 PfRd), since this apo form of PfRd is unfolded and refolded with ease.

We then altered the experiment somewhat, to include B-ME not initially but at a point at which the high temperature had been reached during the very first thermo-chemical perturbation, to obtain essentially the same result. Subsequently, we added the B-ME at various points during the heating and cooling cycles, either before a round of heating or before a round of cooling and discovered that B-ME prevented the occurrence of cold denaturation in PfRd (Figures 7C, 7D, 7E). The data is discussed in the following section.

### Reducing conditions: B-ME accesses its target in cold-denatured PfRd only at temperatures above 65°C (above 85°C, for full reduction) during thermo-chemical perturbation

In the section above, data was presented to clearly establish that the presence of B-ME prevents the occurrence of cold denaturation in PfRd, following thermo-chemical perturbation. Data was also presented to show that even if B-ME is added to protein samples that have already been through one round of thermo-chemical perturbation and cold-denaturation, in the next round the sample displays refolding to native structure, instead of cold-denaturation, presumably owing to the same reducing function of B-ME (now performed at a later stage). This suggests that B-ME reduces something within PfRd, but it is not clear at what temperature this reduction is facilitated by PfRd in its partially cold-denatured TUI or TRI state. In this section, therefore, we explore whether B-ME has unrestricted access (to whatever it is that it reduces) at all temperatures, or whether such access is temperature-dependent, owing to the burial of the target of B-ME's reduction (i.e., either the iron atom, or some disulphide bond) within partially-structured PfRd.

To examine this, B-ME was added to a protein sample that had been taken through only one round of thermo-chemical perturbation and cold-denaturation. The sample was then divided into multiple aliquots and different aliquots were heated up to different temperatures and cooled to room temperature, instead of being heated to 98°C and cooled, as was done previously. We found that if heating was done up to 30, 40, 50 or even 60°C, cooling resulted in cold-denaturation, exactly as if the presence of B-ME were of no consequence at all. On the other hand, when heating was done up to temperatures of 65°C or 70°C, or even higher, prior to cooling, refolding to native structure was observed, instead of the further cold-denaturation seen in the absence of B-ME. This data is summarized in Figure 7F in which far-UV CD spectra collected at different points in this experiment are shown. Further, we found that whereas heating to a temperature of 65°C or 70°C did elicit refolding instead of cold-denaturation, the refolding was not complete. To elicit near-complete refolding, the molecule needed to be heated to a temperature of at least 85°C in the presence of 6 M Gdm.HCl and B-ME, before being cooled to room temperature.

The above data firmly establishes three things: (i) that B-ME performs a reducing function on PfRd; (ii) that the reducing function allows PfRd to display refolding upon cooling after thermo-chemical perturbation (involving partial unfolding), rather than any cold-denaturation of destabilized molecular regions, as is otherwise observed; and (iii) that B-ME can only effectively perform its reducing function upon PfRd if the molecule is exposed to a very high temperature in the presence of 6 M Gdm.HCl (85°C, for complete reduction and refolding). This indicates that the target of B-ME's reduction is buried within PfRd molecules subjected to thermo-chemical perturbation and subsequent cold-denaturation, and that this ‘buried’ target is required to re-surface (to be accessible to B-ME), which is something that only happens at high temperature in the presence of denaturant.

### Reducing conditions: B-ME appears to reduce bound iron within PfRd at high temperature, rather than any disulfides formed through iron's departure

Native PfRd displays some interesting near-UV CD bands owing to the presence of oxidized iron within a particular molecular microenvironment. The iron sulfur cluster of PfRd containing oxidized iron shows a typical spectrum with peaks at 320 nm (−ve), 350 nm(−ve), 400 nm (+ve), 440 nm (+ve), 504 nm (−ve), 560 nm (+ve) and 630 nm (−ve). On the other hand, PfRd containing reduced iron has additional peaks at 315 nm (−ve) and 333 nm (+ve) and reduced intensity of the oxidized peaks [14]. We have already seen that heating of PfRd in the presence of 6M Gdm.HCl to extremely high temperatures (e.g., to 100°C) followed by cooling, leads to cold denaturation.

**Figure 8:**
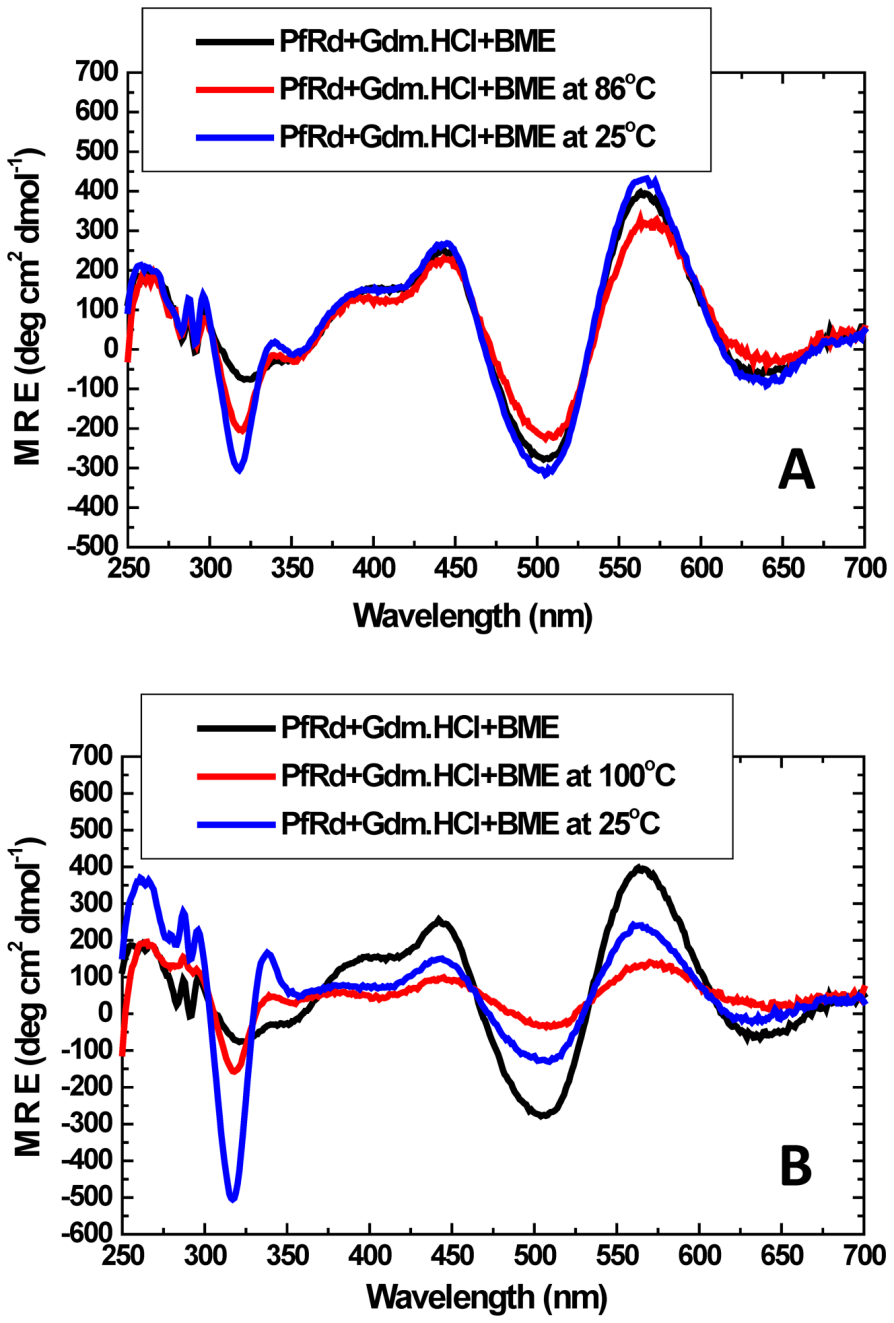
Heating and cooling between 25°C and either 86°C (*Panel* A) or 100°C (*Panel B*), in the presence of B-ME and Gdm.HCl. The negative band at 315-bound iron while other bands mainly represent PfRd-bound iron in the oxidized state. The higher temperature can be seen to provide more access to B-ME to reduce PfRd-bound iron.

When such heating and cooling is done in the presence of B-ME, there is also a gradual reduction in the near-UV CD signal owing to the oxidized iron, at most band positions; concomitantly, a sharp negative CD band is seen to develop at 315 nm, clearly attributable to reduced iron (Figure 8). The 315 nm band is clearly seen in the sample containing denaturant and B-ME at high temperature, and it is evident that when heating is done to the lower temperature of 86°C and the sample is immediately cooled, the bands owing to oxidized iron are not much altered but there is a slight development of a 315 nm band (Figure 8A) presumably owing to the minority population containing reduced bound iron. In contrast, when heating is done up to the higher temperature of 100°C, before cooling, a much stronger negative band at 315 nm is seen and there is also significant reduction in the intensity of the bands owing to oxidized iron (Figure 8B). The behavior of PfRd in the presence of B-ME can thus be clearly seen to be associated with the reduction of the iron in PfRd by B-ME. It is also possible that some of the reduction in intensity of the signal owing to oxidized iron could result from actual physical loss of iron.

We carried out a detailed series of experiments involving the modification of free sulphydryl groups in Cys residues by alkylation through iodoacetamide (IA), and used mass spectrometry to determine whether indeed Cys residues in cold-denatured PfRd remain free or disulphide-bonded. In the Supporting Information in File S1, a separate detailed section is included to describe these multiple types of experiments (with explanations of mass peaks observed, as well as scanned copies of mass spectra). In summary, we found that the Cys residues in cold-denatured PfRd are not accessible to the alkylating agent at room temperature, but at high temperatures the Cys residues are accessible to the alkylating agent and evidence is obtained of alkylated species corresponding to the IA adduction of each of the four Cys residues, in an independent manner, with populations of one, two, three and four Cys residues alkylated by IA. Perhaps the most important experiment in this set is one that shows the following: When PfRd is heated to 100°C in the presence of Gdm.HCl, in the absence of B-ME, and cooling is initiated but halted at 90°C for the addition of IA, it is observed that the IA is able to alkylate all four Cys residues in the protein. However, during the cooling from 100°C to 90°C, evidently cold-denaturation is already occurring, with all four Cys residues free and in non-disulfide bonded condition. Therefore, clearly the cold-denaturation cannot be linked to the formation of any disulphide bonds in PfRD through a ‘cause-effect’ relationship, even if it occurs to some degree.

## Further Discussions and Perspectives

We have used the word ‘*Trishanku*’ to refer to non-native states detected and reported hereIn ancient Indian mythology, *Trishanku* is used to refer to any entity trapped between extreme material and non-material states of existence, and the word would appear to be appropriate to describe hyperthermophile protein conformational states trapped between fully-unfolded and native conformations.

Hyperthermophile proteins are thought to lack the ability to refold from a completely unfolded state (indeed studies of rubredoxin, amongst other proteins, helped to establish this some years ago [14]), and there have been suggestions that some hyperthermophile proteins can probably only fold cotranslationally to native structure [1]. Since the completely unfolded form of a hyperthermophile protein cannot remain completely unfolded after the denaturing condition is removed/reversed (i.e., some chain segments may be expected to fold and bury away hydrophobic surface area, even if not the entire protein), it stands to reason that a non-native, partially-folded state might be obtained through attempts to refold every single irreversibly-unfolded hyperthermophile protein. What is remarkable in the studies reported here, and in our previous report on PfuTIM [1], however, is that we show that there is not one single non-native state/structure feasibly generated by the polypeptide chain of a hyperthermophile protein, but rather virtually as many conformers as one cares to create through differential treatments of the kind demonstrated here, involving forced cold denaturation, both in course of chain unfolding and refolding. What is even more remarkable, perhaps, is that fact that this should be the case with such a small protein as rubredoxin (PfRd). Clearly, the lack of observation of any inter-conversion amongst conformers in these studies establishes that the conformational state of the rubredoxin chain is profoundly under kinetic control, and that the molecule unfolds ‘bit-by-bit’, even as has been argued before [5],[9]. As we have previously dwelt upon the mechanisms by which such ‘bit-by-bit’ unfolding might operate [1], and the connections between such unfolding and the kinetic stability of hyperthermophile proteins, we shall not discuss this much further here but refer the reader to our earlier paper describing the similar observations we made with PfuTIM [1]. However, we would like to draw special attention to the kinetic stability of the various different conformers (TUIs and TRIs) generated. It would appear that it is not just the native state of PfRd which is highly kinetically stable as has been reported and discussed previously, [5],[6],[15],[16],[17],[18] but rather that different parts of PfRd are themselves also significantly kinetically stable from the viewpoint of thermal destabilization. Indeed, this reduced cooperativity of different regions of the protein could explain why unfolding does not occur in a facile manner.

We would also like to draw special attention to certain aspects of the implications of what we have shown here. Cold-denaturation is not normally seen in hyperthermophile proteins because their structures are so kinetically-stable. Whereas with most mesophile proteins, cold denaturation is seen upon cooling the protein to a temperature that is a few tens of degrees Centigrade below the normal functioning temperature of the protein (sometimes with the aid of high hydrostatic pressures), with hyperthermophile proteins cold denaturation had never been reported before we showed that it can be made to occur in PfuTIM [1]. Hyperthermophile proteins, which normally function at 100–105°C, are not ordinarily found to be ‘cold-denatured’ at 37°C, although reasoning suggests that they should be cold-denatured if one holds them to be analogous to mesophile proteins. The reason that hyperthermophile proteins ordinarily do not show cold-denaturation is presumably because their native structures are so kinetically-stable (and so slow to undergo changes) that these proteins are unable to respond to lower temperatures and display cold-denaturation as thermodynamic considerations would dictate, on any observable timescale, just as they are unable to respond to higher temperatures and display any heat-denaturation. In fact, hyperthermophile proteins can even be produced in folded form within *E.coli* at 37°C, whereas they would ordinarily be expected to fail to fold at these temperatures (unless they were caused to form under kinetic control, e.g., using folding pathways employing structure-formation during translation on ribosomes, to subsequently remain kinetically-stabilized).

**Figure 9:**
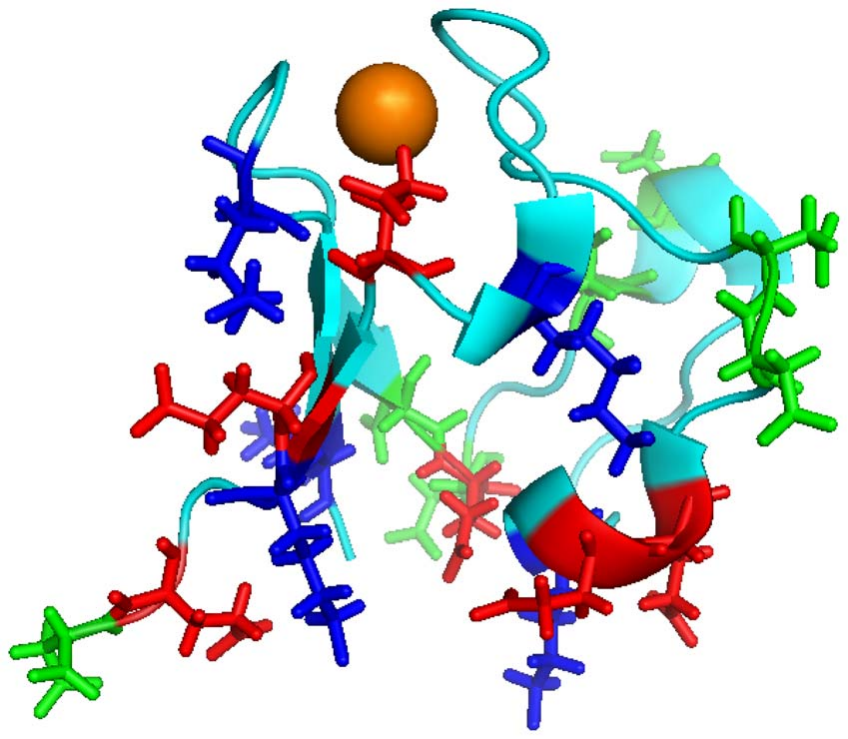
A ribbon-diagram structural representation of holo-PfRd showing the bound iron atom (orange sphere), as well as five surface lysine residue (blue sidechains; K2, K6, K28, K45, K50), seven surface aspartate residues (green sidechains; D13, D15, D18, D20, D34, D35, D53) and six surface glutamate residues (red sidechains : E14, E30, E31, E47, E49, E52).

We reckon that specific interactions occur within hyperthermophile proteins which essentially destroy their capability for undergoing cooperative unfolding, such that this becomes what effectively slows unfolding down so tremendously that one sees no cold-denaturation at 25°C or 37°C. What are these interactions? Both with PfuTIM [1] and now with PfRd, we have established that these could be surface electrostatic interactions, since we have demonstrated that a combination of temperature-changes and a denaturant which is an electrolyte (like Gdm.HCl) unlocks some of the surface salt-bridge interactions, allowing those parts of the protein to now show ‘normal’ cold-denaturation behavior, like any mesophile protein. For reference, we refer the reader to Figure 9 which shows the known structure of the 53 residues-long PfRd (PDB ID : 1VCX) as a ribbon diagram, with its 18 charged surface residues (5 lysines, 7 aspartates and 6 glutamates) forming various salt-bridge interactions. We aver that the use of temperature-changes and Gdm.HCl breaks these salt-bridge interactions in some order. Consequently, different sections of PfRd in which these interactions have been destroyed lose their autonomous stabilization, and unfolding can occur locally in a much more cooperative manner, e.g., involving a loop region in one location, or a beta-strand in another location.

Therefore, we aver that the obtaining of multiple conformations is primarily due to the fact that the hyperthermophile protein is kinetically stabilized – through the lowering of its ability for undergoing cooperative unfolding – because of the greater independence of unfolding amongst different regions of the protein, in part, facilitated by numerous surface salt-bridge interactions which can be individually destroyed by structural perturbations involving the charged partner residues – since this allows some parts of the protein to be unfolded, while other parts remain folded. How does cold-denaturation fit into all this? The obtaining of cold-denaturation – occurring to different extents in different populations - is a consequence of the differential unlocking of these salt-bridge interactions, allowing structure-loss to occur upon cooling in certain parts of the protein, while in other parts of the protein structure-loss is not allowed to occur because of the retention of surface salt-bridges in those regions. Thus, what we show is that cold-denaturation is seen progressively as the kinetically-stabilized hyperthermophile protein is turned into something more-and-more akin to a mesophile protein, as it loses all those special interactions which increase structural autonomy of its parts and destroy the cooperative behavior of the whole.

Of course, in PfuTIM, versus PfRd, the exact mechanisms of kinetic stabilization could differ (and indeed do appear to differ somewhat). In PfuTIM, the increased autonomy of stabilization of beta-alpha units (by surface salt-bridges) appears to destroy cooperativity. In PfRd, the iron-binding dependent formation of a specific packing of aromatic sidechains in a particular scheme in a cluster in the protein's interior appears to be responsible for destroying cooperativity (by creating a subtly different structure in which specific salt-bridges are allowed to differentially stabilize different parts of the protein). This is supported by the fact that when PfRd folds into a subtly different structure in the absence of any iron, that structure shows none of the peculiar behavior of a hyperthermophile protein. In fact, PfRd refolded without iron is just like any ordinary mesophile protein [8],[13]. The cold-denaturation and heat renaturation behavior of hyperthermophile proteins is satisfying because it reveals that the only reason that these proteins do not ordinarily show cold-denaturation is because they unfold very slowly – whether they are being cooled, or heated!

Another point worth noting is that the iron-sulphur cluster in PfRd cannot be held to be entirely responsible for its high kinetic stability, although it clearly affects how the molecule behaves during cooling after thermo-chemical perturbation, especially in relation to its redox status which seems to determine whether the iron atom becomes the nucleus around which a structural core remains formed in PfRd (yielding, and exposing the iron atom to redox agents only above temperatures exceeding 65–70°C). Our data shows that TRI-II (which does not have the cluster) is also non-native; therefore, all aspects of PfRd's behavior cannot be attributed to the iron-sulphur cluster. It has been shown earlier that apo-PfRd has nearly the same structure as holo-PfRd at temperatures below those effecting denaturation [8], such that folding of PfRd without the iron-sulphur cluster would have been expected to take it to the native state at 25°C, if apo-PfRd were itself under purely thermodynamic, and not kinetic, control.

In a separate paper (not yet in print; unpublished observations), we have established that (a) PfRd folded in the absence of iron has a different packing of six aromatic sidechains in its interior from the packing of the same sidechains in holo-PfRd containing a bound iron atom, and (b) removal of the iron atom from folded holo-PfRd does not cause it to change conformation. Thus, apo-PfRd forms that have been produced through folding in the absence of iron, or through removal of iron from folded PfRd, differ profoundly, with the latter apo-form mimicking holo-PfRd in almost every respect, including the possession of hyperthermal stability. As already mentioned, it is known that PfRd folded in the absence of iron shows little kinetic stability, or hyperthermal stability [8], behaving just like any ordinary mesophile protein of moderate stability, whereas holo-PfRd is one of the most hyperthermally stable proteins known to man. We find that the role of the binding of the iron atom is to force PfRd to fold into a conformation incorporating a subtly different packing of aromatic side chains in the protein's interior, which is primarily responsible for its hyperthermal stability, with subsequent removal of the iron atom from the folded structure (without effecting any unfolding) leaving the protein unaltered. These observations reconcile our statement that the iron atom's oxidation status is important, with our contention that the subtle structural (packing) details influenced by iron-binding are ultimately more important than the presence of the iron atom itself. Our data suggests that the influence of the reducing agent, beta-mercaptoethanol, on the cold-denaturation behavior of PfRd largely involves the oxidation state of the iron atom, and not any adventitious disulfide bonding.

We would like to stress here though that although we have used the word intermediate in connection with the non-native states (TUIs and TRIs) detected and reported here, in no way do we intend to indicate that these so-called ‘intermediates’ are on-pathway structural ensembles that are, in any manner, associated with the unfolding and refolding of PfRd.

Finally, we wish to point out this may be one of the few proteins of such a small size which remains monomeric in all its non-native states, such that these non-native conformational intermediates do not have to be confused with products of aggregation-linked pathways that hold up unfolding, or refolding. As such, NMR-based conformational studies of the TUIs and TRIs reported here, and of the many other TUIs and TRIs that may be generated by further changing the treatment of PfRd, could provide a fascinating picture of what the PfRd chain does in terms of structure formation. We intend to carry out such studies ourselves, and hope that others will do so too.

## Supporting Information

File S1 **Supporting information and figures. Figure S1, Production details, protein construct details, isoelectric point, molar extinction coefficient, amino acid sequence, and SDS-PAGE profile of purified PfRd. Figure S2, DNA sequencing electrophoretogram of PfRd, showing with arrows the boundaries marking the restriction sites Nde I and Xho I, used for cloning the PfRd-encoding gene into the pET23-a vector. Figure S3, TRI data. Figure S4, TRI data. Figure S5, TRI data. Figure S6, Time-trace of PfRd in 6 M Gdm.HCl at 100°C (top panel). Figure S7, Time-trace of PfRd in 6 M Gdm.HCl at 20°C (top panel).**
(DOCX)
