# Supplementary material for: Hyperthermophile Protein Behavior: Partially-Structured Conformations of Pyrococcus furiosus Rubredoxin Monomers Generated through Forced Cold-Denaturation and Refolding
Source: PLoS One. 2014 Mar 6;9(3):e80014. doi: 10.1371/journal.pone.0080014 (PMC3945965; doi:10.1371/journal.pone.0080014)
Supplement: File S1 — Supporting information and figures. Figure S1, Production details, protein construct details, isoelectric point, molar extinction coefficient, amino acid sequence, and SDS-PAGE profile of purified PfRd. Figure S2, DNA sequencing electrophoretogram of PfRd, showing with arrows the boundaries marking the restriction sites Nde I and Xho I, used for cloning the PfRd-encoding gene into the pET23-a vector. Figure S3, TRI data. Figure S4, TRI data. Figure S5, TRI data. Figure S6, Time-trace of PfRd in 6 M Gdm.HCl at 100°C (top panel). Figure S7, Time-trace of PfRd in 6 M Gdm.HCl at 20°C (top panel). (DOCX) [file pone.0080014.s001.docx]

**SUPPORTING INFORMATION**

**Chandrayan et al. “Hyperthermophile protein behavior : Partially-structured, ….”**

**Figure S1 :** Production details, protein construct details, isoelectric point, molar extinction coefficient, amino acid sequence, and SDS-PAGE profile of purified PfRd.


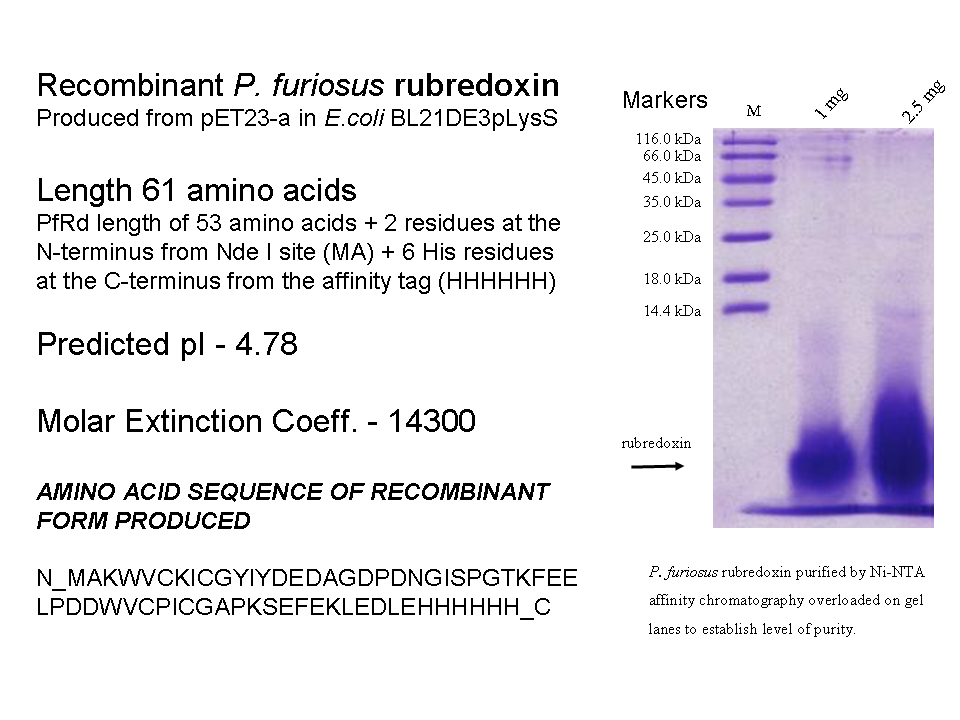


**Figure S2** : DNA sequencing electrophoretogram of PfRd, showing with arrows the boundaries marking the restriction sites Nde I and Xho I, used for cloning the PfRd-encoding gene into the pET23-a vector.


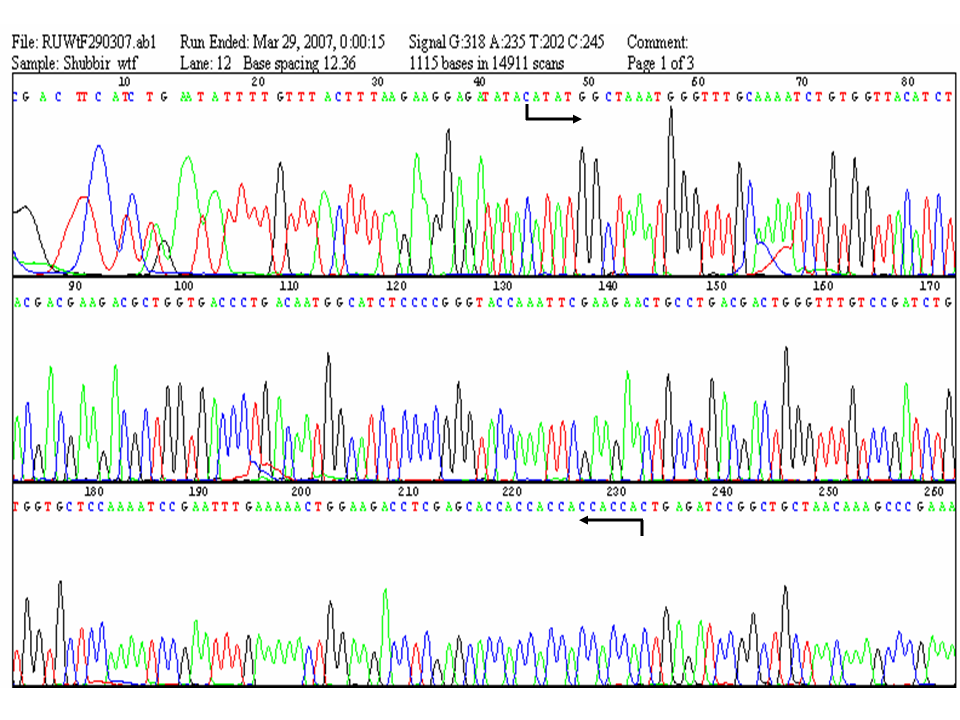


**Figure S3** : TRI data. MALDI-TOF mass spectra collected at different time points (0, 30 and 60 minutes) during subtilisin-treatment of native PfRd (1^st^ column of three panels), TRI-I (2^nd^ column of three panels) and TRI-II (3^rd^ column of three panels).


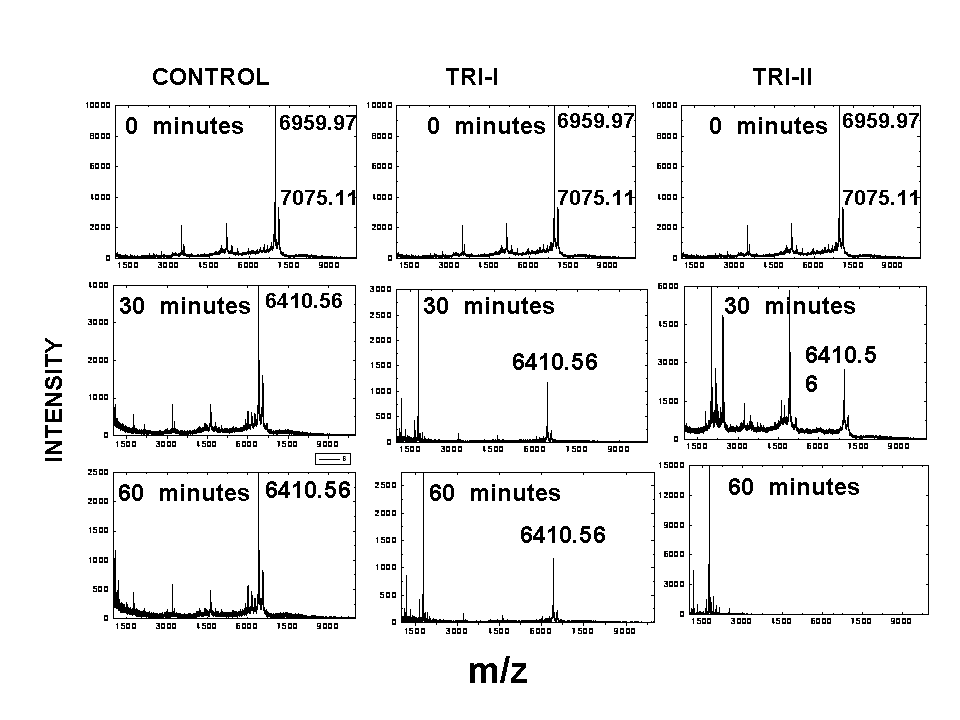


**Figure S4** : TRI data. MALDI-TOF mass spectra collected at different time points (0, 30 and 60 minutes) during trypsin-treatment of native PfRd (1^st^ column of three panels), TRI-I (2^nd^ column of three panels) and TRI-II (3^rd^ column of three panels).


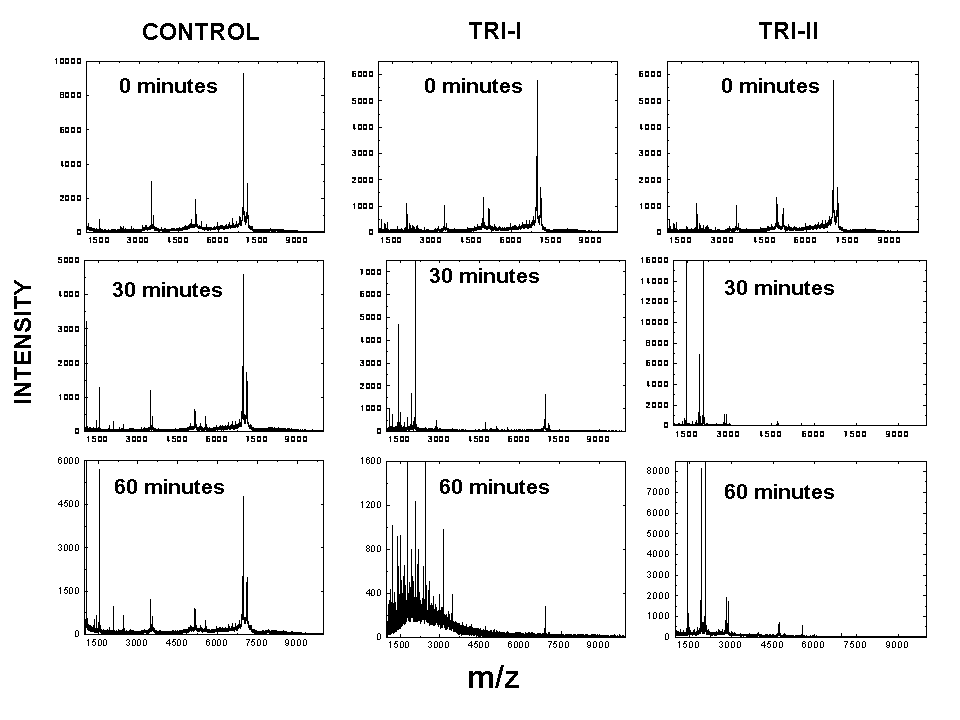


**Figure S5** : TRI data. Monitoring of the mean residue ellipticity CD signal at 222 nm of TRI-I (*Panel A*) and TRI-II (*Panel B*) as function of increasing (black curve) and decreasing (red curve) temperatures, and of the fluorescence emission of 10 μM ANS in the absence (control) and presence of TRI-I and TRI-II (0.25 mg/ml).


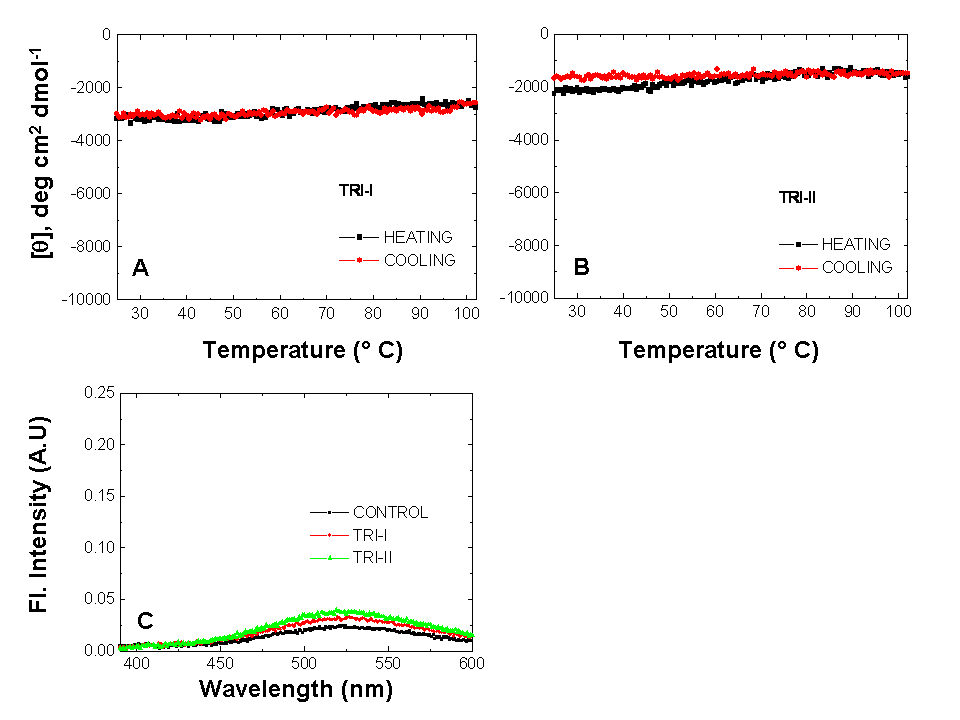


**Figure S6** : Time-trace of PfRd in 6 M Gdm.HCl at 100 °C (top panel). Far-UV CD spectra collected at various time-points during the above experiment (bottom panel).


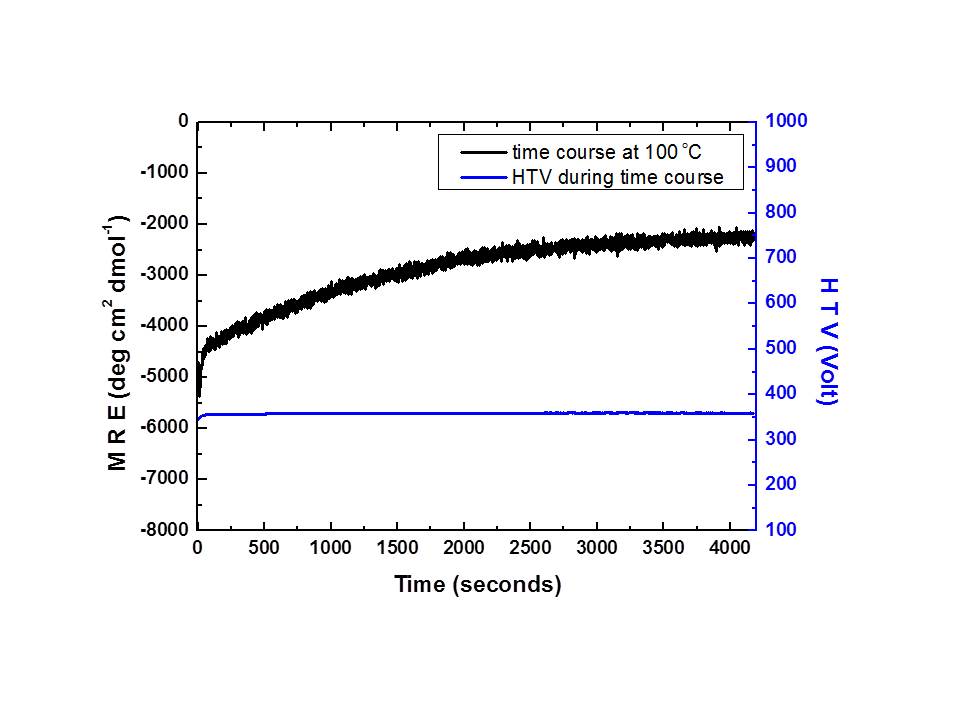


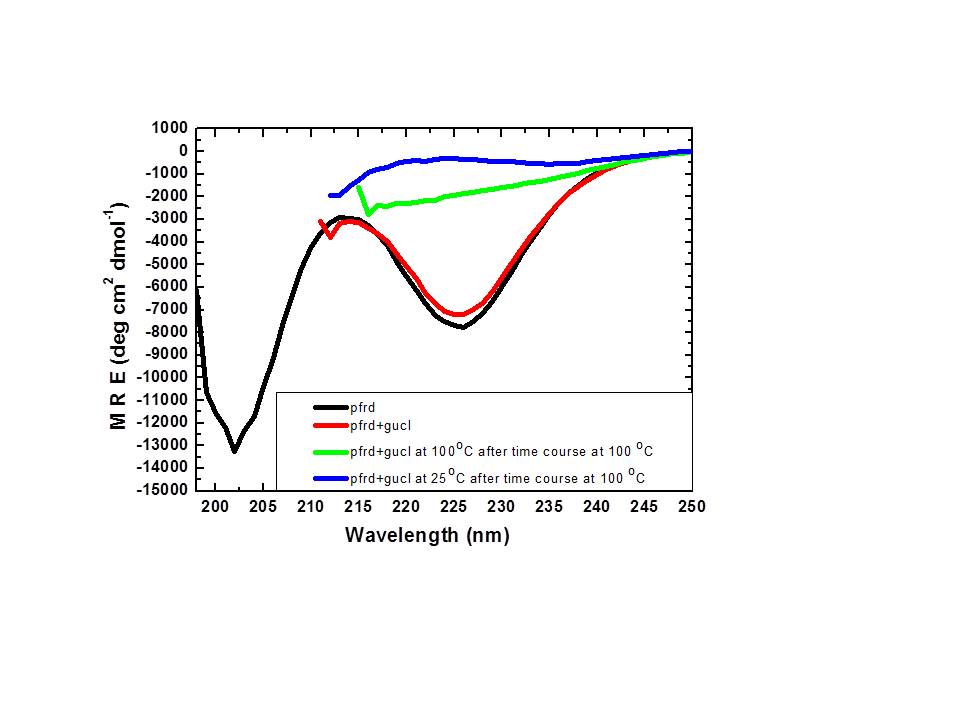


**Figure S7** : Time-trace of PfRd in 6 M Gdm.HCl at 20 °C (top panel). Far-UV CD spectra collected at various time-points during the above experiment (bottom panel).


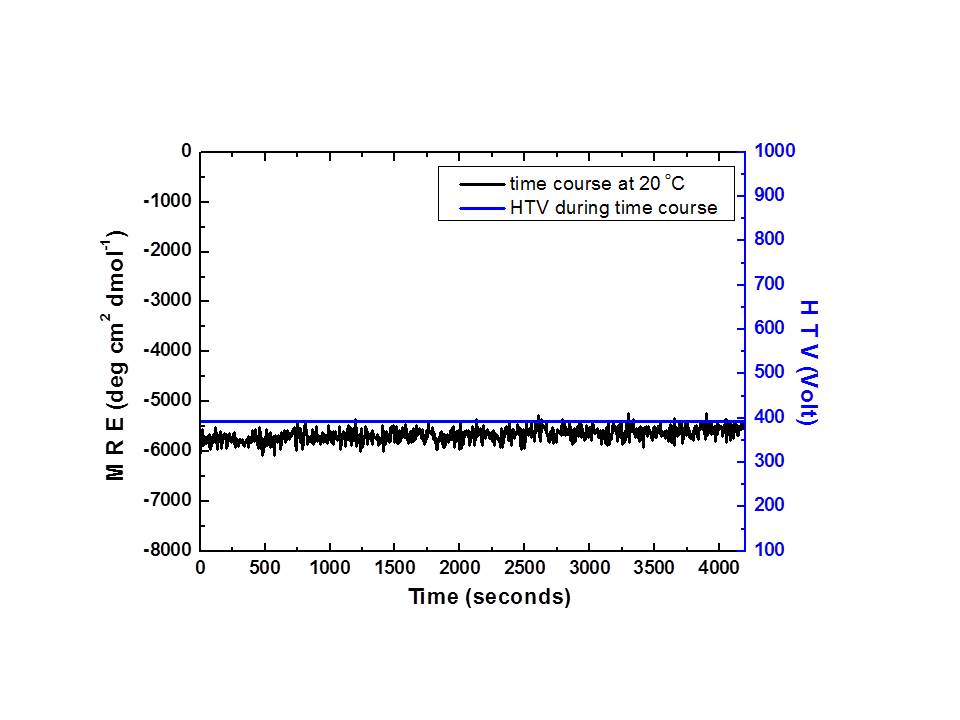


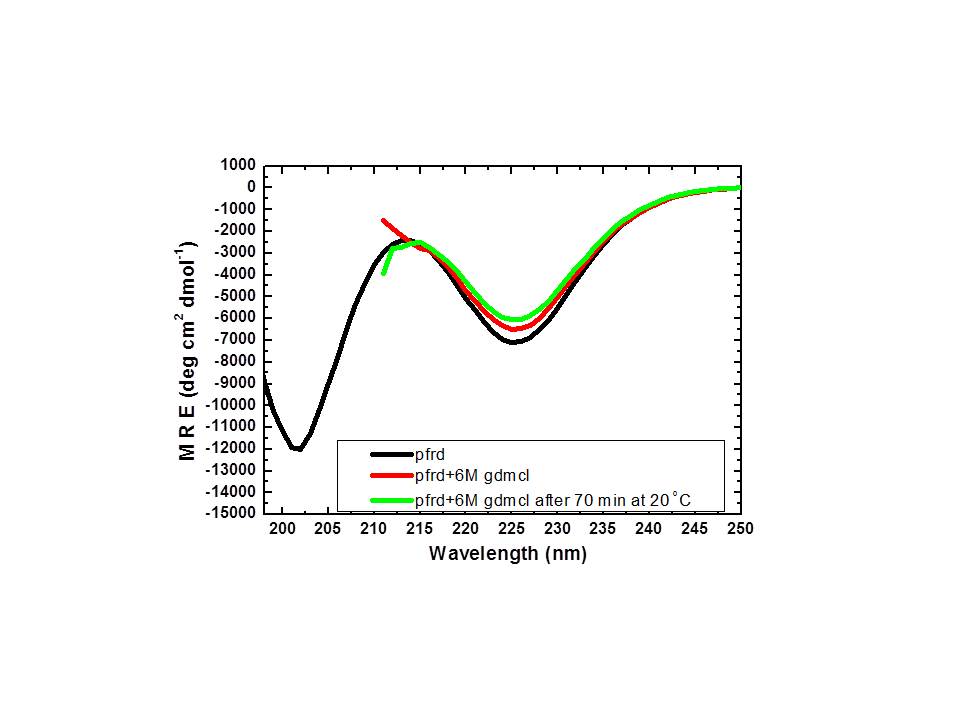


*SCREENING OF DISULFIDE PRESENCE IN VARIOUS STATES OF PfRD*

In most of the protocols, iodoacetamide, in presence and absence of a reducing agent, is used as cysteine modifying agent. Modified cysteines are detected using mass spectrometric analysis by increase of 57 Da over cysteine(s) with respect to the control peak(s). Here also, in all the experiments, iodoacetamide (IA, Sigma-A3221) was used as cysteine modifying (alkylation) agent and 2-mercaptoethanol was used as reducing agent. IAA powder was dissolved in 600 µL milliQ water to give 500 mM stock concentration. The working concentrations of IAA and 2-mercaptoethanol used were 40 mM and 10 mM respectively. The buffer used was 20 mM sodium phosphate buffer, pH 7.2. PfRd has four cysteines bound to iron. In PfRd, a hyperthermophile protein, cysteines are only accessible only at high temperature (data not shown) in presence of 6M Gdm.HCl. To modify the cysteines, PfRd was incubated with IAA and 2-mercaptoethanol (presence or absence, as required) at 90 °C or above for 10 minutes in the presence of 6M Gdm.HCl (20 mM sodium phosphate buffer, pH 7.2). The resulting solution was desalted using reverse phase Zip-Tip_C18_ tips (Millipore) and eluted samples were analysed using MALDI-TOF, using a Voyager DE STR (AB Sciex) mass spectrometer.

*Detection of disulphides in cold denaturation of PfRd:*

All samples had 0.5 mg/ml of PfRd and 6M Gdm.HCl in final concentrations. Two types of samples were made- **S1**: PfRd and 6M Gdm.HCl with no heat treatment, **S2**: PfRd and 6M Gdm.HCl which was heated upto 100 °C and cooled back to 25 °C. Heating and cooling of samples was done in Eppendorf, Mastercycler (pro S) at a ramp rate of ~4°C/ minute. S1 were used to detect the disulphide formation during first heating and cooling cycle while S2 were used to detect disulphides already formed during one heating and cooling cycle. For detection of disulphides during and after cold denaturation a series of experiments were done which had different steps of thermal and chemical treatments. Chemical treatments were given in final concentrations as given above. These experiments are summarized in the table shown below (actual MALDI data not shown, but available, for each reaction product). Temperatures, at which the chemical treatments were done, are given in parentheses in each step. After elution from Zip-Tip_C18_ tips the samples were analysed by MALDI-TOF.

| **Exp** | **S1/S2 (step 1)** | **step 2** | **step 3** | **step 4** | **step 5** | **step 6** | **No. of IA bound** |
| --- | --- | --- | --- | --- | --- | --- | --- |
| 1. | PfRd | No heating | No incubation | No cooling or addition | No incubation | No further cooling or incubation | 0 |
| 2. | S1+bme+ **IA**  (25°C) | Heat  90 °C | 10 min.  90 °C | Cool  25 °C |  |  | 4 |
| 3 | S1+ **IA**  (25°C) | Heat  90 °C | 10 min.  90 °C | Cool  25 °C |  |  | 4 |
| 4. | S1+bme  (25°C) | Heat  100 °C | Cool  90 °C | Add  **IA** | 10 min.  90 °C | Cool  25 °C | 4 |
| 5. | S1+bme  (25°C) | Heat  100 °C | Cool  90 °C | ------ | 10 min.  90 °C | Cool  25 °C | NA |
| 6. | S1  (25°C) | Heat  100 °C | Cool  90 °C | Add  **IA** | 10 min.  90 °C | Cool  25 °Cg | Mainly 3 and 4, but also 1, 2 |
| 7. | S1  (25°C) | Heat  100 °C | Cool  70 °C | Add  **IA** | 10 min.  70 °C | Cool  25 °C | Mainly 0 but also 1,2,3 |
| 8. | S1 + bme  (25°C) | Heat  100 °C | Cool  70 °C | Add  **IA** | 10 min.  70 °C | Cool  25 °C | 4 |
| 9. | S2+bme+ **IA**  (25°C) | Heat  90 °C | 10 min.  90 °C | Cool  25 °C |  |  | 4 |
| 10. | S2+ bme  (25°C) | Heat  90 °C | 10 min.  90 °C | Cool  25 °C |  |  | NA |
| 11. | S2+ **IA**  (25°C) | Heat  90 °C | 10 min.  90 °C | Cool  25 °C |  |  | Mainly 0 but also 1,2,3,4 |

**Results and Discussion:**

1. *No disulphides are formed during first heating:*

MALDI-TOF spectra of PfRd in presence and absence of 6M Gdm.HCl and 2-mercaptoethanol (bme) show similar peak of ~7293 Da (Fig A, E, H) as control molecular weight. Heating after adding IA to sample S1 (fresh PfRd + 6M Gdm.HCl), both in presence and absence of 2-mercaptoethanol, shows similar increase of masses (57 ^x^ 4=228 Da) over all four cysteines. The increments correspond to one IA molecule bound to all four cysteines.

1. *No disulphides are formed during first cooling after first heating:*

Treating Sample S1 with IA at 90 °C during cooling after first heating gives species having all cysteines bound to IA both in the presence and in the absence of 2-mercaptoethanol. In the similar experiments, when IA treatment without 2-mercaptoethanol was given at 70 °C rather than 90 ° C, a mixed population bound to 1, 2 and 3 IA molecules was obtained. On the other hand, if 2-mercaptoethanol was present from start and similar treatment of IA was given at 70 °C, only population bound to 4 IA was found.

1. *No disulphides are formed in cold denatured form:*

IA treatment to sample S2 (PfRd+ 6M Gdm.HCl, heated and cooled) in presence of 2-mercaptoethanol resulted in IA bound to all cysteines. IA treatment in absence of 2-mercaptoethanol also resulted in the populations having 3 and 4 IA bound.
